# Supplementary material for: Mapping cerebral blood perfusion and its links to multi-scale brain organization across the human lifespan
Source: PLoS Biol. 2025 Jul 29;23(7):e3003277. doi: 10.1371/journal.pbio.3003277 (PMC12324687; doi:10.1371/journal.pbio.3003277)
Supplement: S1 Fig — Each dot corresponds to a participant’s mean blood perfusion level within the white matter mask defined as part of the HCP ASL preprocessing pipeline (male: blue, female: red). Top: Sex-stratified generalized additive models for location, scale and shape (GAMLSS) are used to model age-related changes in blood perfusion across the human lifespan. Bottom: Statistical comparisons of white matter blood perfusion between males and females are conducted within each age bin (13–15 years: t = −3.76, p=2.62×10^−4^; 15–17 years: t = −4.97, p=3.43×10^−6^; 19–21 years: t = −4.73, p=1.36×10^−5^; 21–23 years: t = −2.96, p=5.29×10^−3^; 35–47 years: t = −7.26, p=1.75×10^−11^; 47–57 years: t = −6.69, p=9.47×10^−11^; 57–67 years: t = −3.86, p=1.88×10^−4^; 67–77 years: t = −3.39, p=9.98×10^−4^; >77 years: t = −4.61, p=1.01×10^−5^; other age bins: p > 0.05). (PDF) [file pbio.3003277.s001.pdf]

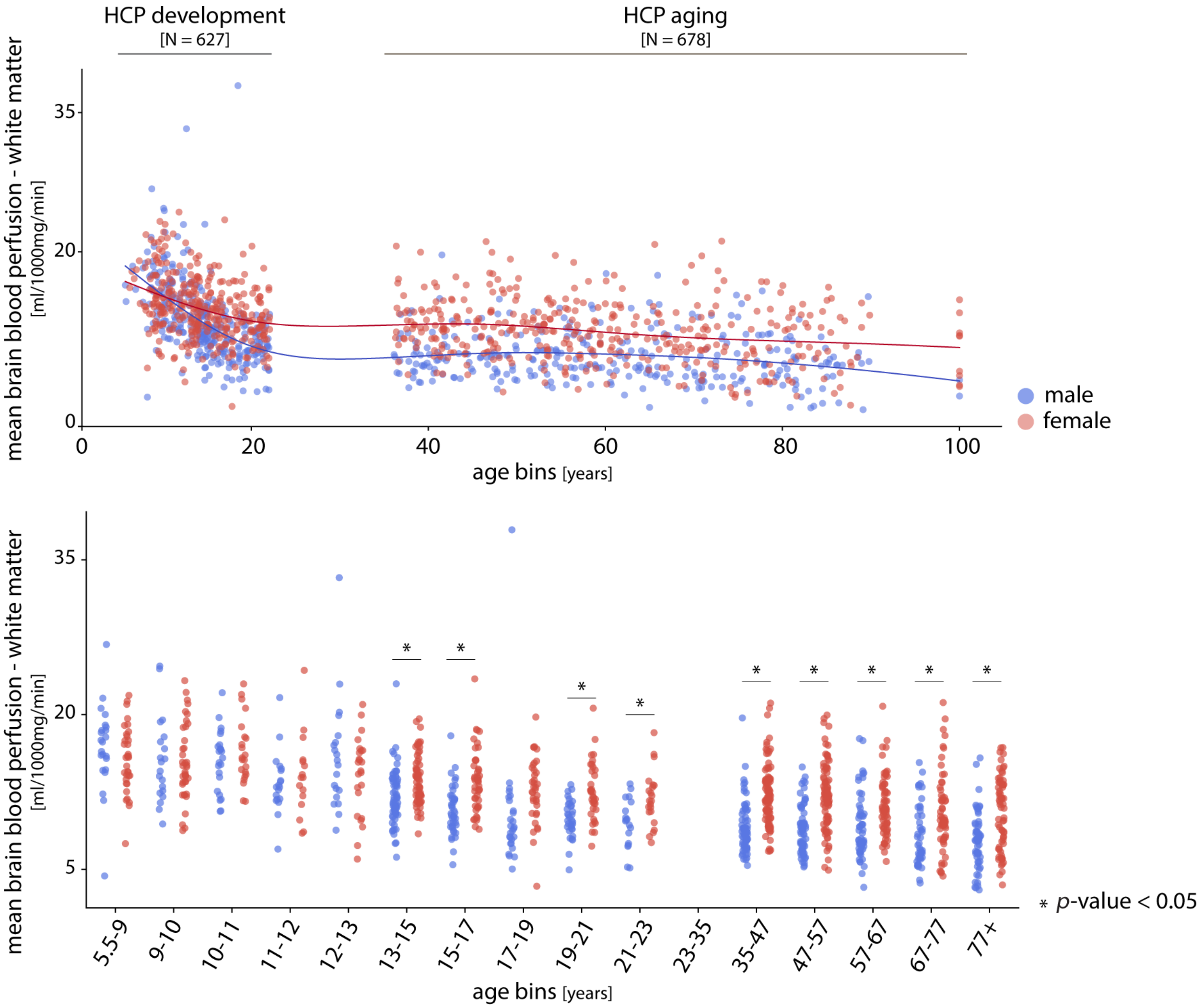

Figure S1. **Mapping white matter blood perfusion across participants |** Each dot corresponds to a participant's mean blood perfusion level within the white matter mask defined as part of the HCP ASL preprocessing pipeline (male: blue, female: red). Top: Sex-stratified generalized additive models for location, scale and shape (GAMLSS) are used to model age-related changes in blood perfusion across the human lifespan. Bottom: Statistical comparisons of white matter blood perfusion between males and females are conducted within each age bin (13–15 years: $t = -3.76$, $p = 2.62 \times 10^{-4}$; 15–17 years: $t = -4.97$, $p = 3.43 \times 10^{-6}$; 19–21 years: $t = -4.73$, $p = 1.36 \times 10^{-5}$; 21–23 years: $t = -2.96$, $p = 5.29 \times 10^{-3}$; 35–47 years: $t = -7.26$, $p = 1.75 \times 10^{-11}$; 47–57 years: $t = -6.69$, $p = 9.47 \times 10^{-11}$; 57–67 years: $t = -3.86$, $p = 1.88 \times 10^{-4}$; 67–77 years: $t = -3.39$, $p = 9.98 \times 10^{-4}$; > 77 years: $t = -4.61$, $p = 1.01 \times 10^{-5}$; other age bins: $p > 0.05$).
