# Supplementary material for: Mapping cerebral blood perfusion and its links to multi-scale brain organization across the human lifespan
Source: PLoS Biol. 2025 Jul 29;23(7):e3003277. doi: 10.1371/journal.pbio.3003277 (PMC12324687; doi:10.1371/journal.pbio.3003277)
Supplement: S2 Fig — (a) The vertex/voxel-wise mean blood perfusion maps across all participants (HCP-D and HCP-A), stratified by biological sex. Maps are shown on the inflated and 2D flat cortical surfaces (fsLR) and on a T2-weighted group-average template [290]. (b) Left: Perfusion is higher in female brain compared to the male brain (t = 9.27, ptwo-sided=7.21×10^−20^). Each dot corresponds to a participant’s whole brain blood perfusion (male: blue, female: red). Right: Correlation between female (x-axis) and male mean blood perfusion (y-axis) (r = 0.99). Each dot corresponds to mean blood perfusion for a vertex (grey) or a voxel (black). The unity line of x = y is shown as a reference. (PDF) [file pbio.3003277.s002.pdf]

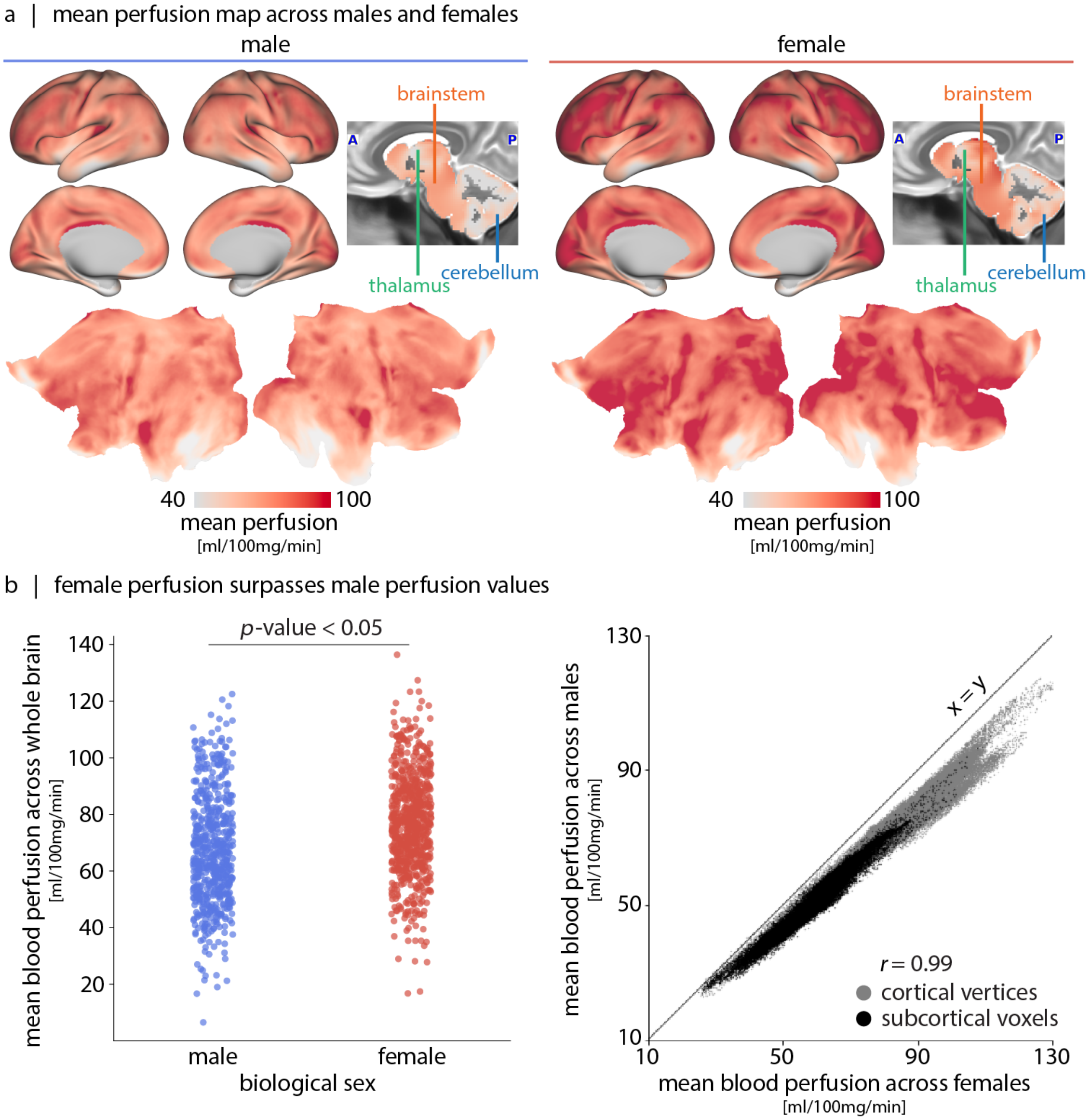

Figure S2. **Sex-difference in cerebral blood perfusion |** (a) The vertex/voxel-wise mean blood perfusion maps across all participants (HCP-D and HCP-A), stratified by biological sex. Maps are shown on the inflated and 2D flat cortical surfaces (fsLR) and on a T2-weighted group-average template [1]. (b) Left: Perfusion is higher in female brain compared to the male brain ($t = 9.27$, $p_{\text{two-sided}} = 7.21 \times 10^{-20}$). Each dot corresponds to a participant's whole brain blood perfusion (male: blue, female: red). Right: Correlation between female (x-axis) and male mean blood perfusion (y-axis) ($r = 0.99$). Each dot corresponds to mean blood perfusion for a vertex (grey) or a voxel (black). The unity line of $x = y$ is shown as a reference.

References

1. Glasser MF, Smith SM, Marcus DS, Andersson JL, Auerbach EJ, Behrens TE, et al. The human connectome project's neuroimaging approach. *Nature Neuroscience*. 2016;19(9):1175–1187.
