# Supplementary material for: Mapping cerebral blood perfusion and its links to multi-scale brain organization across the human lifespan
Source: PLoS Biol. 2025 Jul 29;23(7):e3003277. doi: 10.1371/journal.pbio.3003277 (PMC12324687; doi:10.1371/journal.pbio.3003277)
Supplement: S3 Fig — There exists lower blood perfusion in subcortical voxels compared to the cortical vertices in both male and female participant groups (male: t = 22.96, ptwo-sided=1.25×10^−96^; female: t = 30.65, ptwo-sided=3.96×10−159). Each dot corresponds to the mean cortical perfusion (grey) or mean subcortical perfusion per participant (black). (PDF) [file pbio.3003277.s003.pdf]

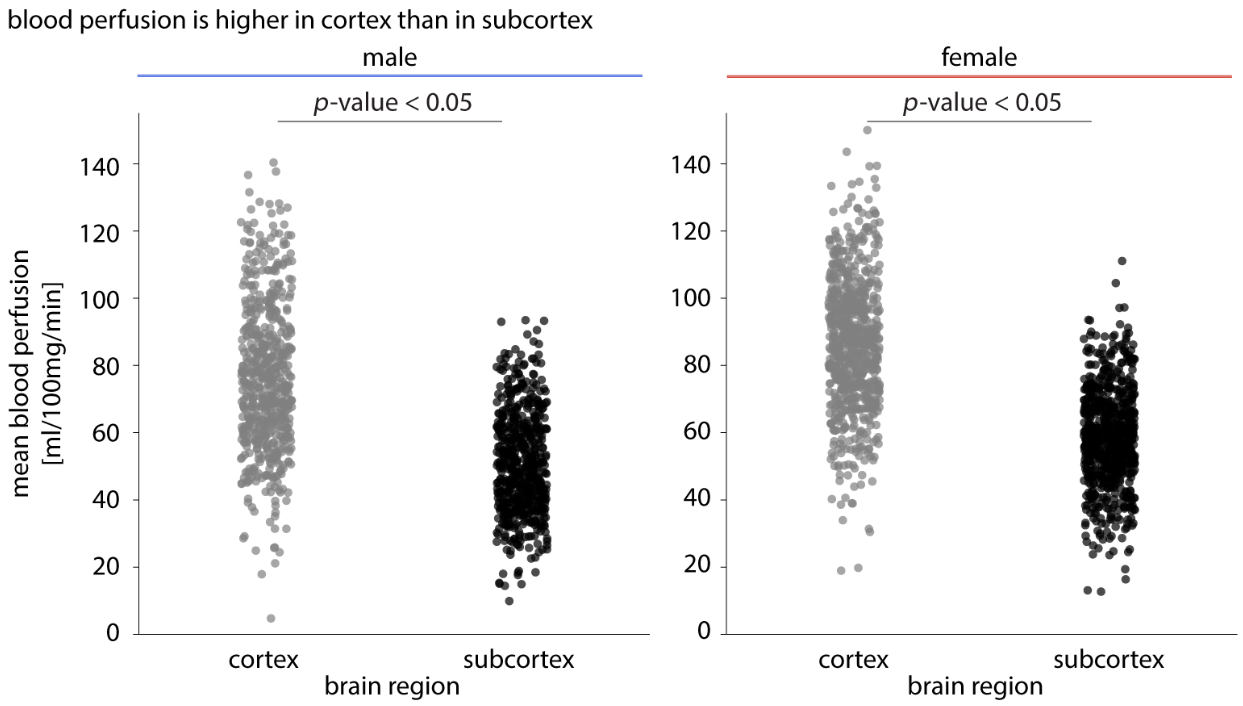

Figure S3. **Blood perfusion is higher in cortical versus the subcortical tissue |** There exists lower blood perfusion in subcortical voxels compared to the cortical vertices in both male and female participant groups (male: $t = 22.96$, $p_{\text{two-sided}} = 1.25 \times 10^{-96}$; female: $t = 30.65$, $p_{\text{two-sided}} = 3.96 \times 10^{-159}$). Each dot corresponds to the mean cortical perfusion (grey) or mean subcortical perfusion per participant (black).
