# Supplementary material for: Mapping cerebral blood perfusion and its links to multi-scale brain organization across the human lifespan
Source: PLoS Biol. 2025 Jul 29;23(7):e3003277. doi: 10.1371/journal.pbio.3003277 (PMC12324687; doi:10.1371/journal.pbio.3003277)
Supplement: S4 Fig — (a) First principal component loadings are shown per participant (male: blue, female: red). PC loadings assess the similarity between individual participants’ cerebral blood perfusion pattern and the composite map captured by the PC. (b) Individual-level Pearson correlation values between each participant’s perfusion map and their FC strength map (male: blue, female: red). Here, FC strength for a region is defined as the absolute weighted sum of all edges connected to that region. In the developmental window (5–22 years), coupling between FC strength and regional perfusion develops (ρ=0.09, ppermutation=2.60×10^−2^, 1000 repetitions); and in the aging window (36–100 years) decoupling between the two happens (ρ=−0.24, ppermutation=9.99×10^−4^, 1000 repetitions). (c) Correlation between average cortical perfusion (y-axis) and average absolute functional connectivity strength (x-axis) (r = 0.26). The significance of the correlation is assessed via permutation testing (ppermutation=9.99×10^−4^, 1000 repetitions). Each dot represents data from an individual participant, with colors corresponding to their age. (PDF) [file pbio.3003277.s004.pdf]

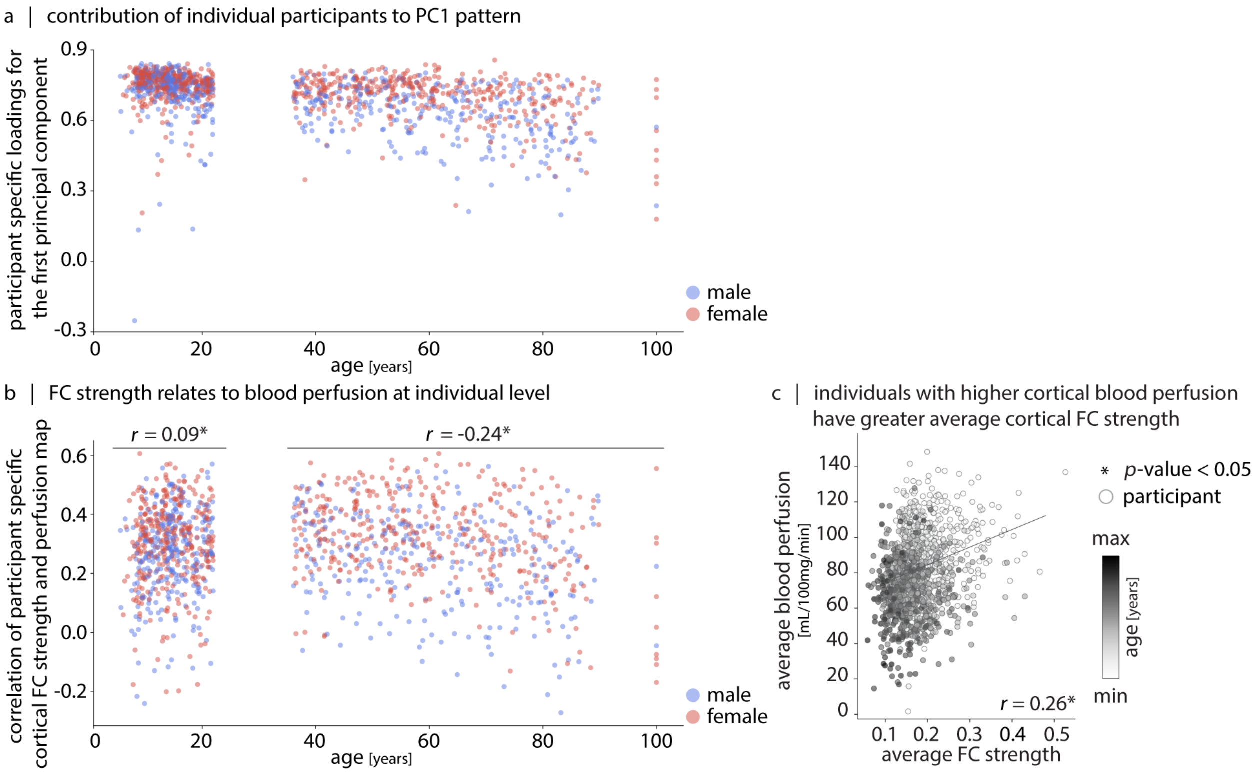


Figure S4. **Cortical blood perfusion participant loadings and the score map relevance to functional connectivity (FC) strength |** (a) First principal component loadings are shown per participant (male: blue, female: red). PC loadings assess the similarity between individual participants' cerebral blood perfusion pattern and the composite map captured by the PC. (b) Individual-level Pearson correlation values between each participant's perfusion map and their FC strength map (male: blue, female: red). Here, FC strength for a region is defined as the absolute weighted sum of all edges connected to that region. In the developmental window (5–22 years), coupling between FC strength and regional perfusion develops ($\rho = 0.09$, $p_{\text{permutation}} = 2.60 \times 10^{-2}$, $1\,000$ repetitions); and in the aging window (36–100 years) decoupling between the two happens ($\rho = -0.24$, $p_{\text{permutation}} = 9.99 \times 10^{-4}$, $1\,000$ repetitions). (c) Correlation between average cortical perfusion ($y$-axis) and average absolute functional connectivity strength ($x$-axis) ($r = 0.26$). The significance of the correlation is assessed via permutation testing ($p_{\text{permutation}} = 9.99 \times 10^{-4}$, $1\,000$ repetitions). Each dot represents data from an individual participant, with colors corresponding to their age.
