# Supplementary material for: Mapping cerebral blood perfusion and its links to multi-scale brain organization across the human lifespan
Source: PLoS Biol. 2025 Jul 29;23(7):e3003277. doi: 10.1371/journal.pbio.3003277 (PMC12324687; doi:10.1371/journal.pbio.3003277)
Supplement: S5 Fig — (a) Cerebral blood flow map from PET imaging is shown on the inflated and 2D flat cortical surfaces (fsLR). (b) Correlation between the z-scored cerebral blood perfusion from PET data (x-axis) and cerebral blood perfusion score from ASL imaging (y-axis) (r = 0.63; pspin=9.99×10^−4^). Inferior temporal cortex has low blood perfusion in both PET and ASL data, indicating that the low perfusion in this region is not caused by ASL modality-specific signal drop-out. (PDF) [file pbio.3003277.s005.pdf]

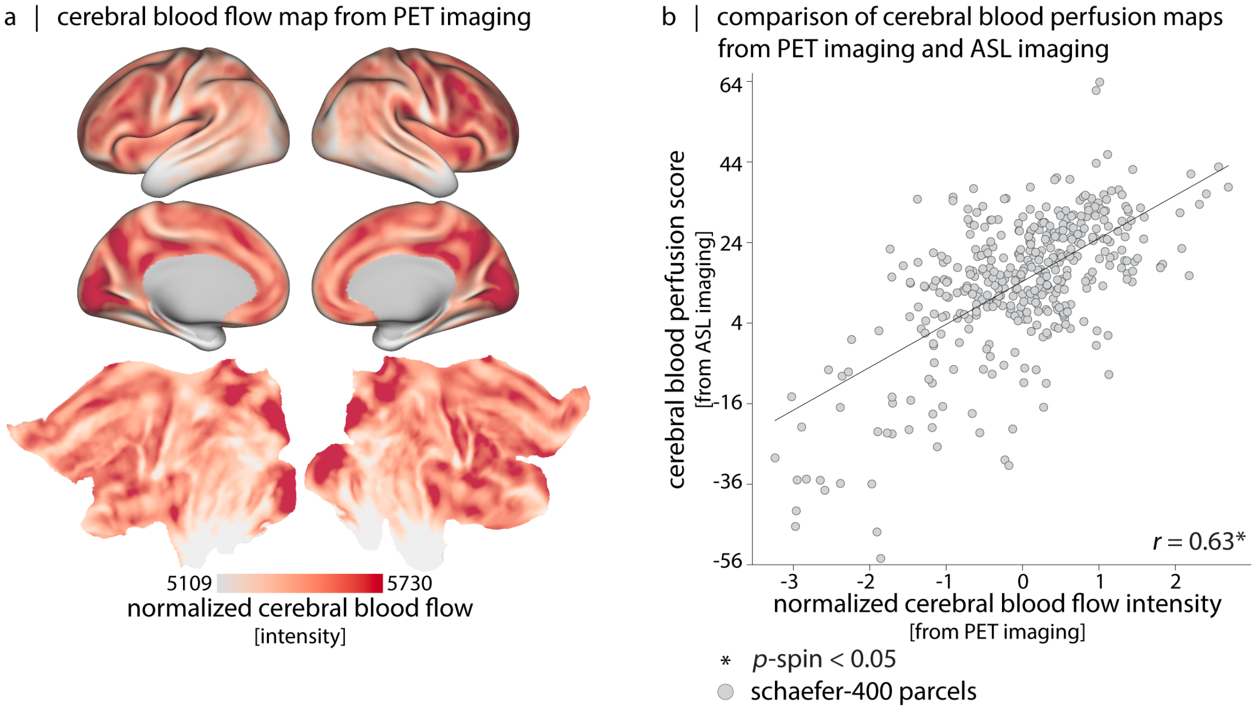

Figure S5. **Comparison of blood perfusion maps from PET and ASL imaging |** (a) Cerebral blood flow map from PET imaging is shown on the inflated and 2D flat cortical surfaces (fsLR). (b) Correlation between the z-scored cerebral blood perfusion from PET data (x-axis) and cerebral blood perfusion score from ASL imaging (y-axis) ($r = 0.63$; $p_{\text{spin}} = 9.99 \times 10^{-4}$). Inferior temporal cortex has low blood perfusion in both PET and ASL data, indicating that the low perfusion in this region is not caused by ASL modality-specific signal drop-out.
