# Supplementary material for: Mapping cerebral blood perfusion and its links to multi-scale brain organization across the human lifespan
Source: PLoS Biol. 2025 Jul 29;23(7):e3003277. doi: 10.1371/journal.pbio.3003277 (PMC12324687; doi:10.1371/journal.pbio.3003277)
Supplement: S6 Fig — We first regress out the linear and non-linear effects of age, sex and their interactions from participants’ cerebral blood perfusion maps using general linear models with the following equation: perfusion=β0 +β1×age+β2×sex+β3 ×sex×age+β4×age2+β5 ×sex×age2+β6×age3+β7 ×sex×age3. Next, we concatenate the cleaned individual data, z-score them and perform PCA on the concatenated data matrix. The first component explains about 72.8% of the variance in the data. (a) The brain score map of the first principal component is shown on lateral and medial views of the inflated and 2D flat cortical surfaces (fsLR); the volumetric part is shown on the sagittal view of the T2-weighted group-average template (MNI152). (b) Participant-specific loadings for PC1 are also shown. (c) The obtained brain score map is highly consistent with the PC map in Fig 2A and 2B. The correlation of the obtained score map (after regressing out the co-variates) with the first score perfusion map (shown in Fig 2A and 2B) is equal to 0.93. (PDF) [file pbio.3003277.s006.pdf]

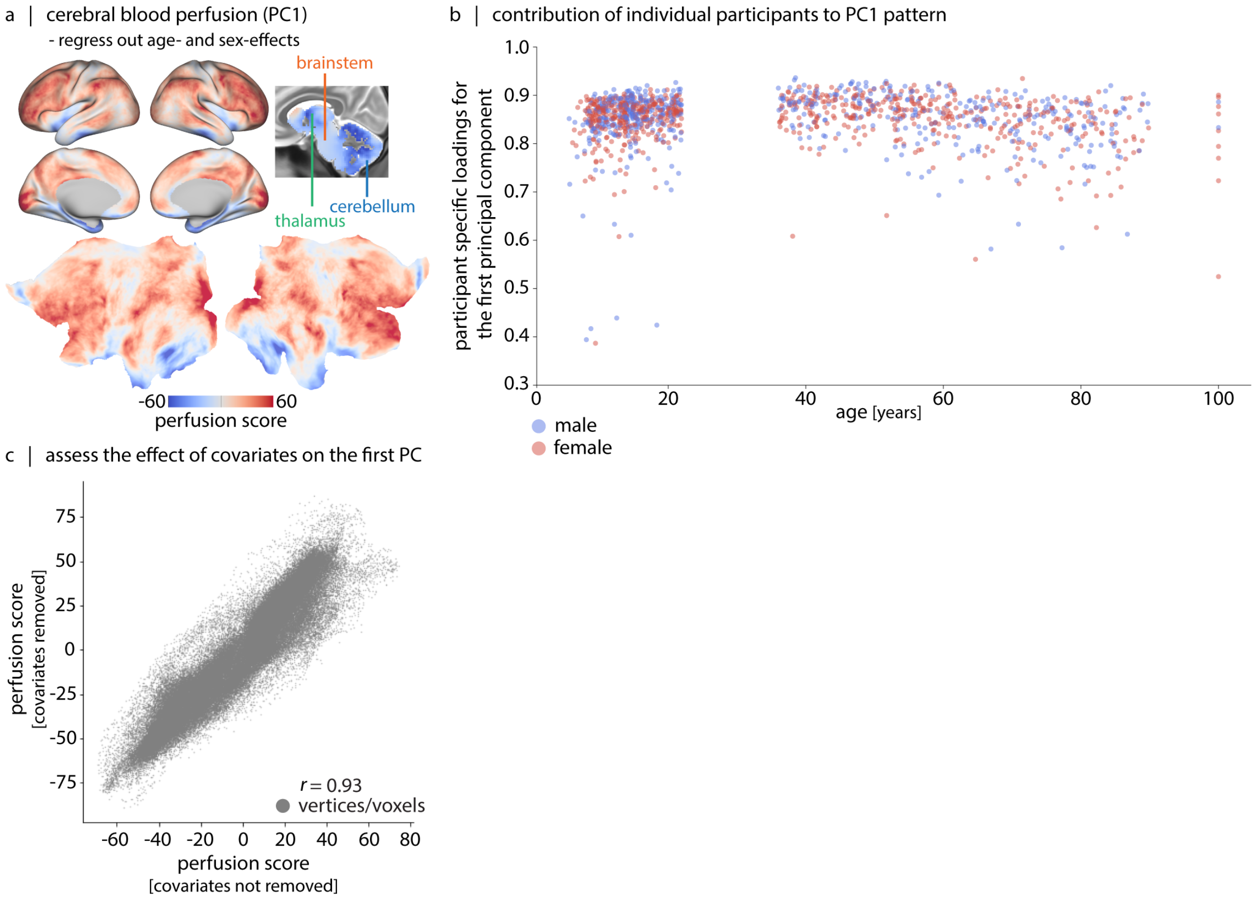

Figure S6. **First principal component of cerebral blood perfusion data after regressing out linear and non-linear effects of sex, age and sex-age interactions |** We first regress out the linear and non-linear effects of age, sex and their interactions from participants' cerebral blood perfusion maps using general linear models with the following equation: perfusion $= \beta_0 + \beta_1 \times$ age $+ \beta_2 \times$ sex $+ \beta_3 \times$ sex $\times$ age $+ \beta_4 \times$ age$^2 + \beta_5 \times$ sex $\times$ age$^2 + \beta_6 \times$ age$^3 + \beta_7 \times$ sex $\times$ age^3. Next, we concatenate the cleaned individual data, z-score them and perform PCA on the concatenated data matrix. The first component explains about 72.8% of the variance in the data. (a) The brain score map of the first principal component is shown on lateral and medial views of the inflated and 2D flat cortical surfaces (fsLR); the volumetric part is shown on the sagittal view of the T2-weighted group-average template (MNI152). (b) Participant-specific loadings for PC1 are also shown. (c) The obtained brain score map is highly consistent with the PC map in Fig 2A and 2B. The correlation of the obtained score map (after regressing out the co-variates) with the first score perfusion map (shown in Fig 2A and 2B) is equal to 0.93.
