# Supplementary material for: Mapping cerebral blood perfusion and its links to multi-scale brain organization across the human lifespan
Source: PLoS Biol. 2025 Jul 29;23(7):e3003277. doi: 10.1371/journal.pbio.3003277 (PMC12324687; doi:10.1371/journal.pbio.3003277)
Supplement: S7 Fig — (a) Mean score value per Tian-S4 subcortical parcel is shown here. Values are sorted from high to low perfusion scores (low perfusion score indicates less cerebral blood flow). (b) The Tian-S4 parcellation is shown on a MNI152 T1-weighted volume space (sagittal and transverse views) [87]; see S3 Table for full parcel names. (PDF) [file pbio.3003277.s007.pdf]

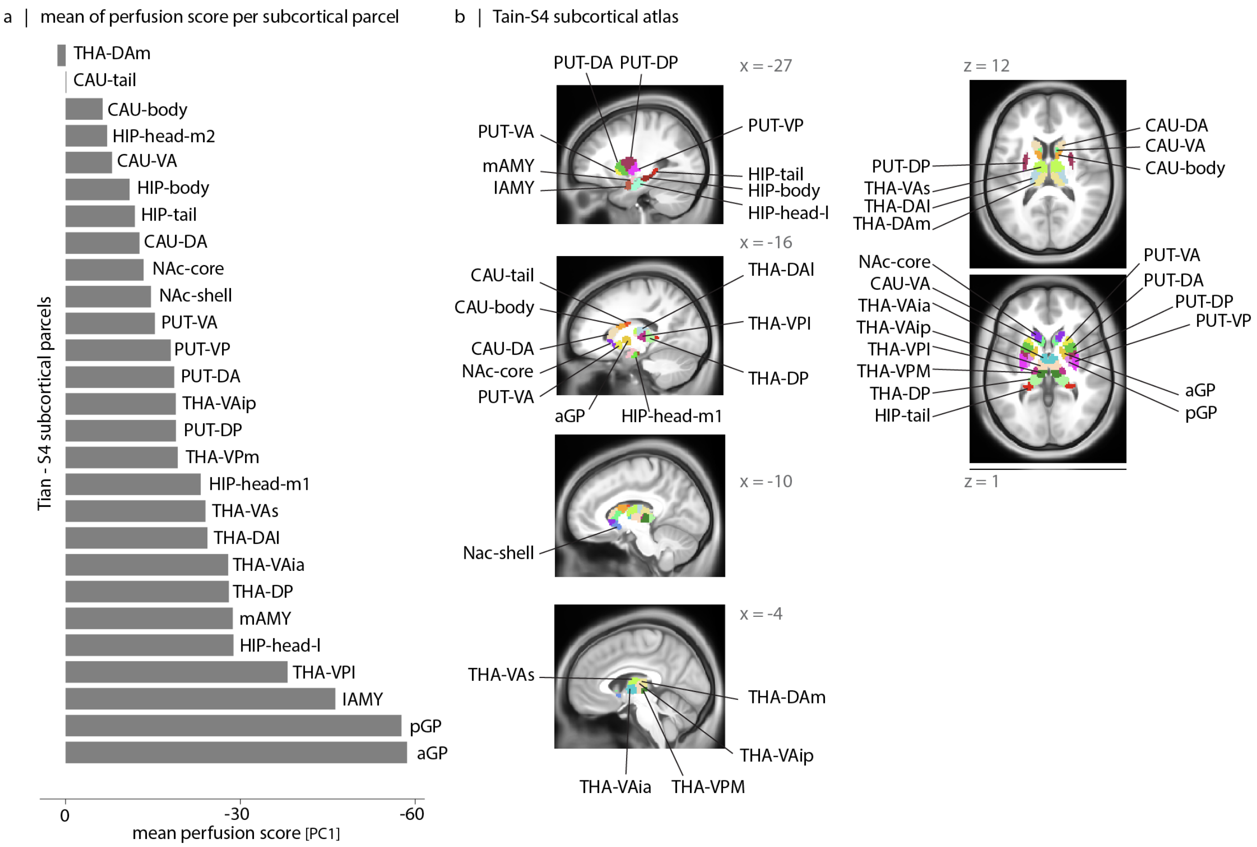

Figure S7. **Quantification of the first perfusion score map at subcortical level |** (a) Mean score value per Tian-S4 subcortical parcel is shown here. Values are sorted from high to low perfusion scores (low perfusion score indicates less cerebral blood flow). (b) The Tian-S4 parcellation is shown on a MNI152 T1-weighted volume space (sagittal and transverse views) [1]; see S3 Table for full parcel names.

References

1. Tian Y, Margulies DS, Breakspear M, Zalesky A. Topographic organization of the human subcortex unveiled with functional connectivity gradients. *Nature Neuroscience*. 2020;23(11):1421–1432.
