# Supplementary material for: Mapping cerebral blood perfusion and its links to multi-scale brain organization across the human lifespan
Source: PLoS Biol. 2025 Jul 29;23(7):e3003277. doi: 10.1371/journal.pbio.3003277 (PMC12324687; doi:10.1371/journal.pbio.3003277)
Supplement: S8 Fig — The top row shows the average z-scored transcriptomic signature of cortical layer I–III, IV, and V–VI, respectively [88]. The bottom row presents scatter plots with perfusion scores on the y-axis and the average z-score of gene maps for each layer on the x-axis. For layer I–III, the transcriptomic signature is defined using 13 genes (C1QL2, C20orf103, CARTPT, DISC1, GLRA3, GSG1L, IGSF11, INPP4B, MFGE8, PVRL3, RASGRF2, SV2C, and WFS1). However, three of these genes (C20orf103, PVRL3, and DISC1) do not meet the differential stability of 0.1 and hence are excluded from the analysis. The transcriptomic signature for layer IV is based on 5 genes (COL6A1, CUX2, TRMT9B, GRIK4, and RORB), all of which pass the differential stability threshold of 0.1. For layer V–VI, there are 28 gene markers (ADRA2A, AKR1C3, ANXA1, B3GALT2, CDH24, CTGF, ETV1, FAM3C, FOXP2, HTR2C, KCNK2, NPY2R, NR4A2, NTNG2, OPRK1, PCDH17, PCDH20, PCP4, PDE1A, RPRM, RXFP1, SNTB1, SYT10, SYT6, TLE4, TOX, TRIB2, and VAT1L). Four of these genes (CDH24, FAM3C, NPY2R, and TRIB2) do not meet the differential stability of 0.1 and are excluded from the analysis. (PDF) [file pbio.3003277.s008.pdf]

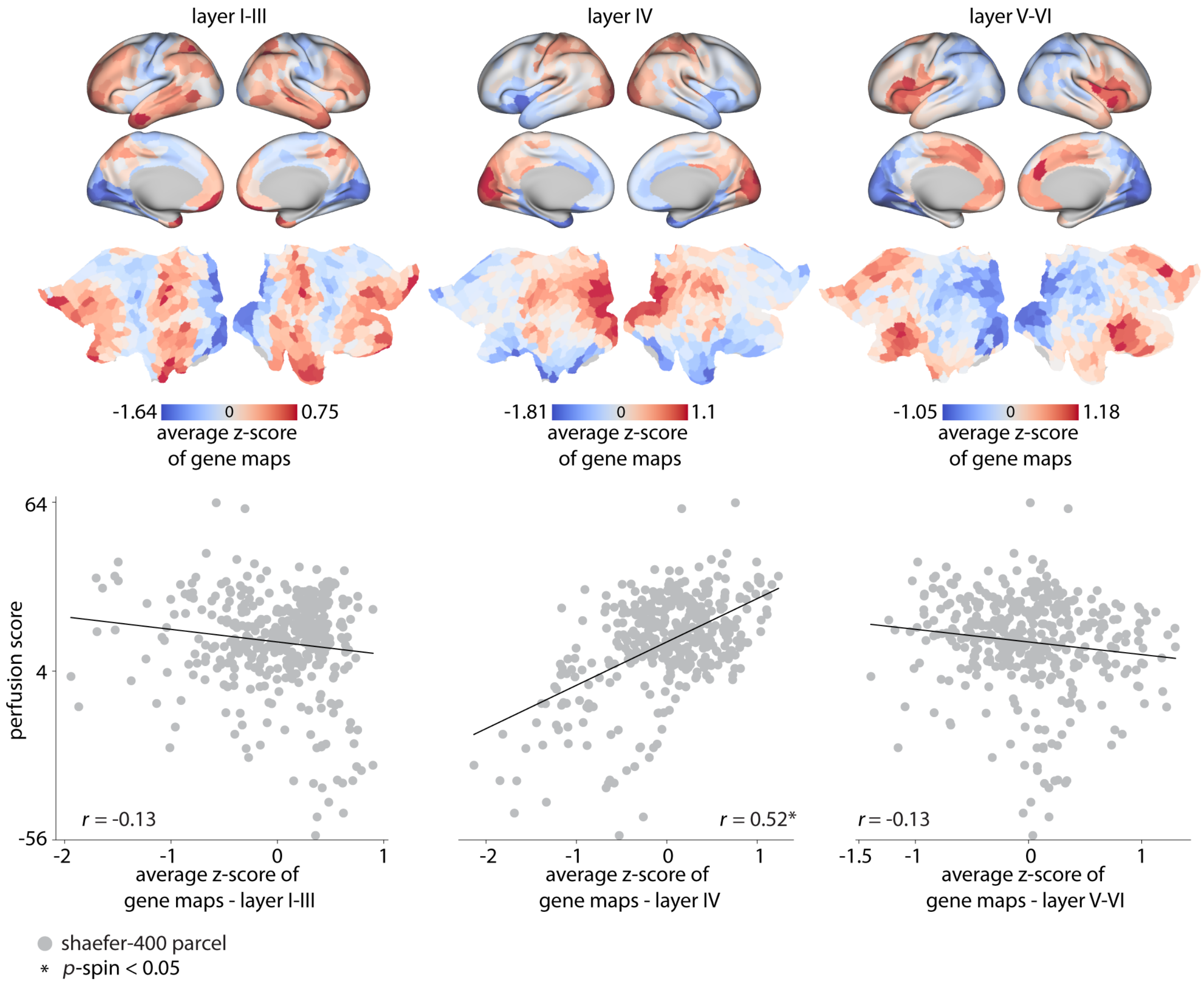

Figure S8. **Relating perfusion and laminar differentiation** | The top row shows the average z-scored transcriptomic signature of cortical layer I–III, IV, and V–VI, respectively [1]. The bottom row presents scatter plots with perfusion scores on the y-axis and the average z-score of gene maps for each layer on the x-axis. For layer I–III, the transcriptomic signature is defined using 13 genes (C1QL2, C20orf103, CARTPT, DISC1, GLRA3, GSG1L, IGSF11, INPP4B, MFGE8, PVRL3, RASGRF2, SV2C, and WFS1). However, three of these genes (C20orf103, PVRL3, and DISC1) do not meet the differential stability of 0.1 and hence are excluded from the analysis. The transcriptomic signature for layer IV is based on 5 genes (COL6A1, CUX2, TRMT9B, GRIK4, and RORB), all of which pass the differential stability threshold of 0.1. For layer V–VI, there are 28 gene markers (ADRA2A, AKR1C3, ANXA1, B3GALT2, CDH24, CTGF, ETV1, FAM3C, FOXP2, HTR2C, KCNK2, NPY2R, NR4A2, NTNG2, OPRK1, PCDH17, PCDH20, PCP4, PDE1A, RPRM, RXFP1, SNTB1, SYT10, SYT6, TLE4, TOX, TRIB2, and VAT1L). Four of these genes (CDH24, FAM3C, NPY2R, and TRIB2) do not meet the differential stability of 0.1 and are excluded from the analysis.

References

1. Burt JB, Demirta¸s M, Eckner WJ, Navejar NM, Ji JL, Martin WJ, et al. Hierarchy of transcriptomic specialization across human cortex captured by structural neuroimaging topography. *Nature Neuroscience*. 2018;21(9):1251–1259.
