# Supplementary material for: Mapping cerebral blood perfusion and its links to multi-scale brain organization across the human lifespan
Source: PLoS Biol. 2025 Jul 29;23(7):e3003277. doi: 10.1371/journal.pbio.3003277 (PMC12324687; doi:10.1371/journal.pbio.3003277)
Supplement: S9 Fig — We use dominance analysis to assess the contribution of biological features to regional variations in cerebral blood perfusion. Dominance analysis distributes the model’s explained variance among the input variables, allowing comparison of their relative contribution to predicting perfusion patterns. (a, b) show results for a model including neurotransmitters, neuropeptides and cortical laminar profiles; and (c, d) show results for a model including only neurotransmitters and neuropeptides. Model fit, expressed as adjusted R2, reaches to 0.64 for the first model (a) and to 0.59 for the second model (c). Asterisks denote significant model fits (in both cases: pspin=9.99×10^−3^, nspin=1000). The percent contributions of each input variable normalized by the total model fit is shown in (b, d). (PDF) [file pbio.3003277.s009.pdf]

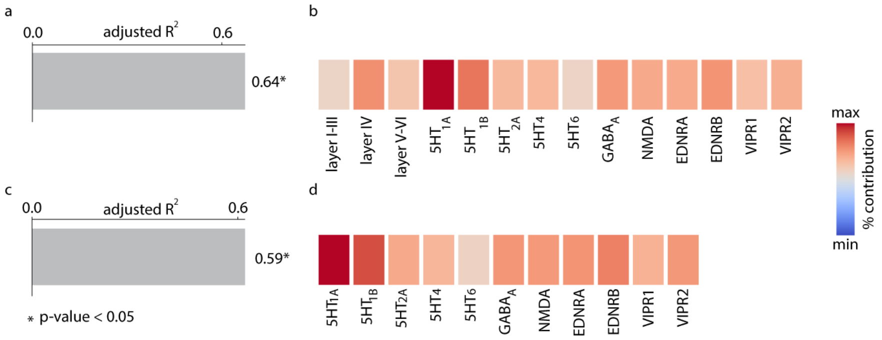

Figure S9. **Relating perfusion to molecular signaling systems and laminar organization |** We use dominance analysis to assess the contribution of biological features to regional variations in cerebral blood perfusion. Dominance analysis distributes the model's explained variance among the input variables, allowing comparison of their relative contribution to predicting perfusion patterns. (a, b) show results for a model including neurotransmitters, neuropeptides and cortical laminar profiles; and (c, d) show results for a model including only neurotransmitters and neuropeptides. Model fit, expressed as adjusted R^2, reaches to 0.64 for the first model (a) and to 0.59 for the second model (c). Asterisks denote significant model fits (in both cases: $p_{\text{spin}} = 9.99 \times 10^{-3}$, $n_{\text{spin}} = 1\,000$). The percent contributions of each input variable normalized by the total model fit is shown in (b, d).
