# Supplementary material for: Mapping cerebral blood perfusion and its links to multi-scale brain organization across the human lifespan
Source: PLoS Biol. 2025 Jul 29;23(7):e3003277. doi: 10.1371/journal.pbio.3003277 (PMC12324687; doi:10.1371/journal.pbio.3003277)
Supplement: S10 Fig — To test the interaction between age and sex on cerebral perfusion during development, we fit a linear model including main effects of age, sex, and their interaction: perfusion=β0+β1×age+β2×sex+β3×age×sex (male = 1, female = 0; β0=112.44). The model reveals age-related decline in perfusion (β1=−1.81, p=2.4×10^−27^). There is also a sex difference in baseline perfusion (β2=8.42, p = 0.026), and an interaction between age and sex (β3=−1.05, p=3.26×10^−5^), suggesting that males exhibit a steeper age-related decline in grayordinates’ perfusion compared to females (male: blue, female: red). (PDF) [file pbio.3003277.s010.pdf]

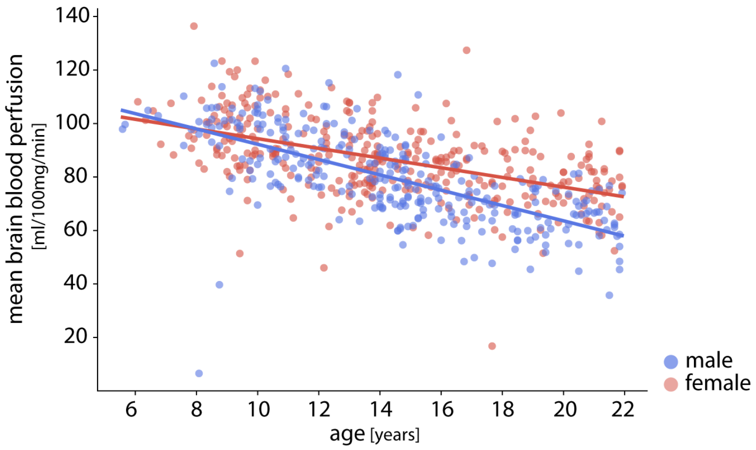

Figure S10. **Sex differences in developmental perfusion trajectories |** To test the interaction between age and sex on cerebral perfusion during development, we fit a linear model including main effects of age, sex, and their interaction: perfusion $= \beta_0 + \beta_1 \times$ age $+ \beta_2 \times$ sex $+ \beta_3 \times$ age $\times$ sex (male $= 1$, female $= 0$; $\beta_0 = 112.44$). The model reveals age-related decline in perfusion ($\beta_1 = -1.81$, $p = 2.4 \times 10^{-27}$). There is also a sex difference in baseline perfusion ($\beta_2 = 8.42$, $p = 0.026$), and an interaction between age and sex ($\beta_3 = -1.05$, $p = 3.26 \times 10^{-5}$), suggesting that males exhibit a steeper age-related decline in grayordinates' perfusion compared to females (male: blue, female: red).
