# Supplementary material for: Mapping cerebral blood perfusion and its links to multi-scale brain organization across the human lifespan
Source: PLoS Biol. 2025 Jul 29;23(7):e3003277. doi: 10.1371/journal.pbio.3003277 (PMC12324687; doi:10.1371/journal.pbio.3003277)
Supplement: S11 Fig — Average age-effect per parcel is shown for HCP-D (a) and HCP-A (b) datasets. (c) Subcortical parcels are defined based on Tian-S4 subcortical parcellation [87]. See S3 Table for full parcel names. (PDF) [file pbio.3003277.s011.pdf]

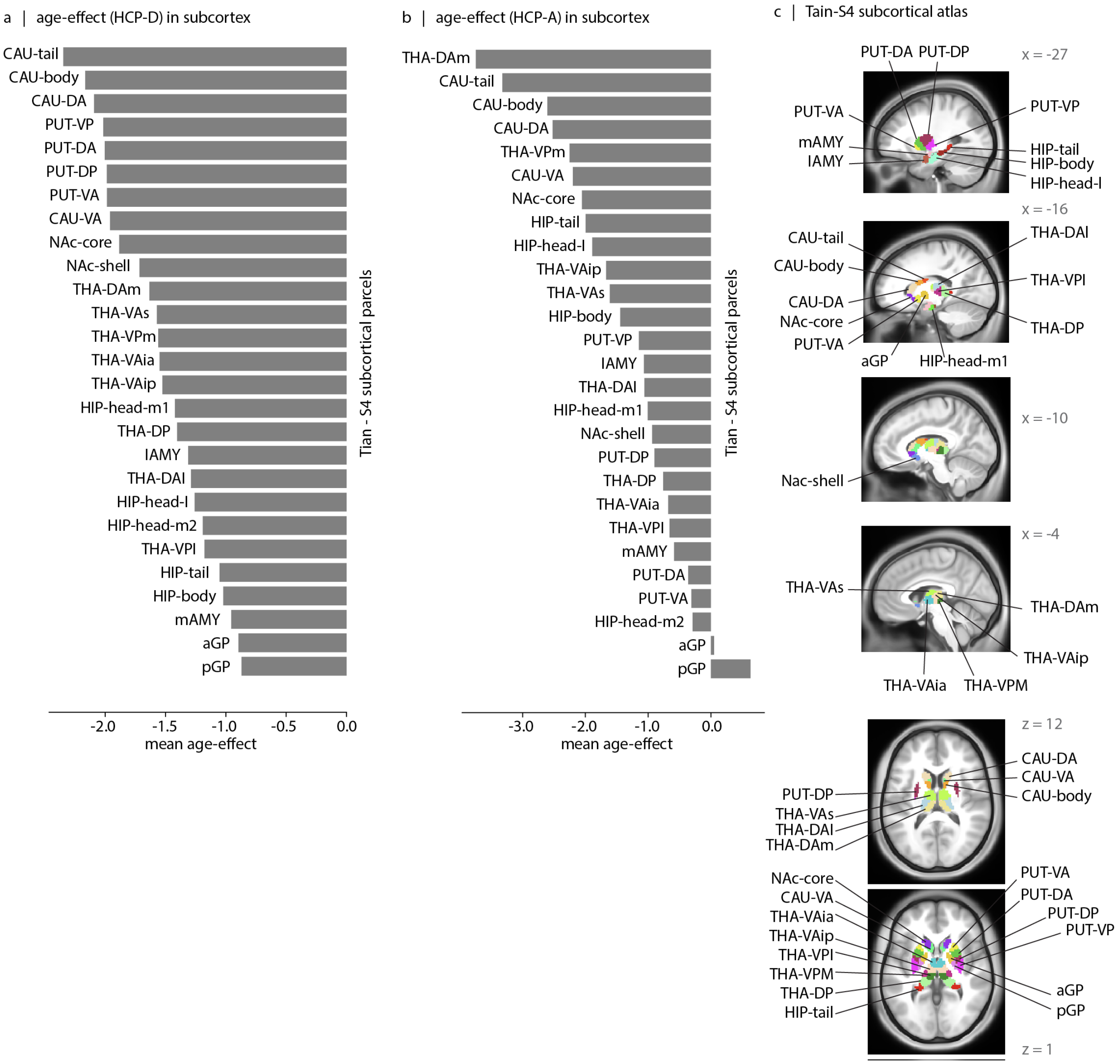

Figure S11. **Mean age-effect in subcortical regions** | Average age-effect per parcel is shown for HCP-D (a) and HCP-A (b) datasets. (c) Subcortical parcels are defined based on Tian-S4 subcortical parcellation [1]. See S3 Table for full parcel names.

References

1. Burt JB, Demirta¸s M, Eckner WJ, Navejar NM, Ji JL, Martin WJ, et al. Hierarchy of transcriptomic specialization across human cortex captured by structural neuroimaging topography. *Nature Neuroscience*. 2018;21(9):1251–1259.
