# Supplementary material for: Mapping cerebral blood perfusion and its links to multi-scale brain organization across the human lifespan
Source: PLoS Biol. 2025 Jul 29;23(7):e3003277. doi: 10.1371/journal.pbio.3003277 (PMC12324687; doi:10.1371/journal.pbio.3003277)
Supplement: S12 Fig — Top: The general linear model (GLM) coefficients for age and biological sex (male = 1, female = 0) are shown on the cortical fsLR surface maps and MNI152 T2-weighted volume space. Bottom: Areas with Bonferroni-corrected p-values below 0.05 are marked in red. (PDF) [file pbio.3003277.s012.pdf]

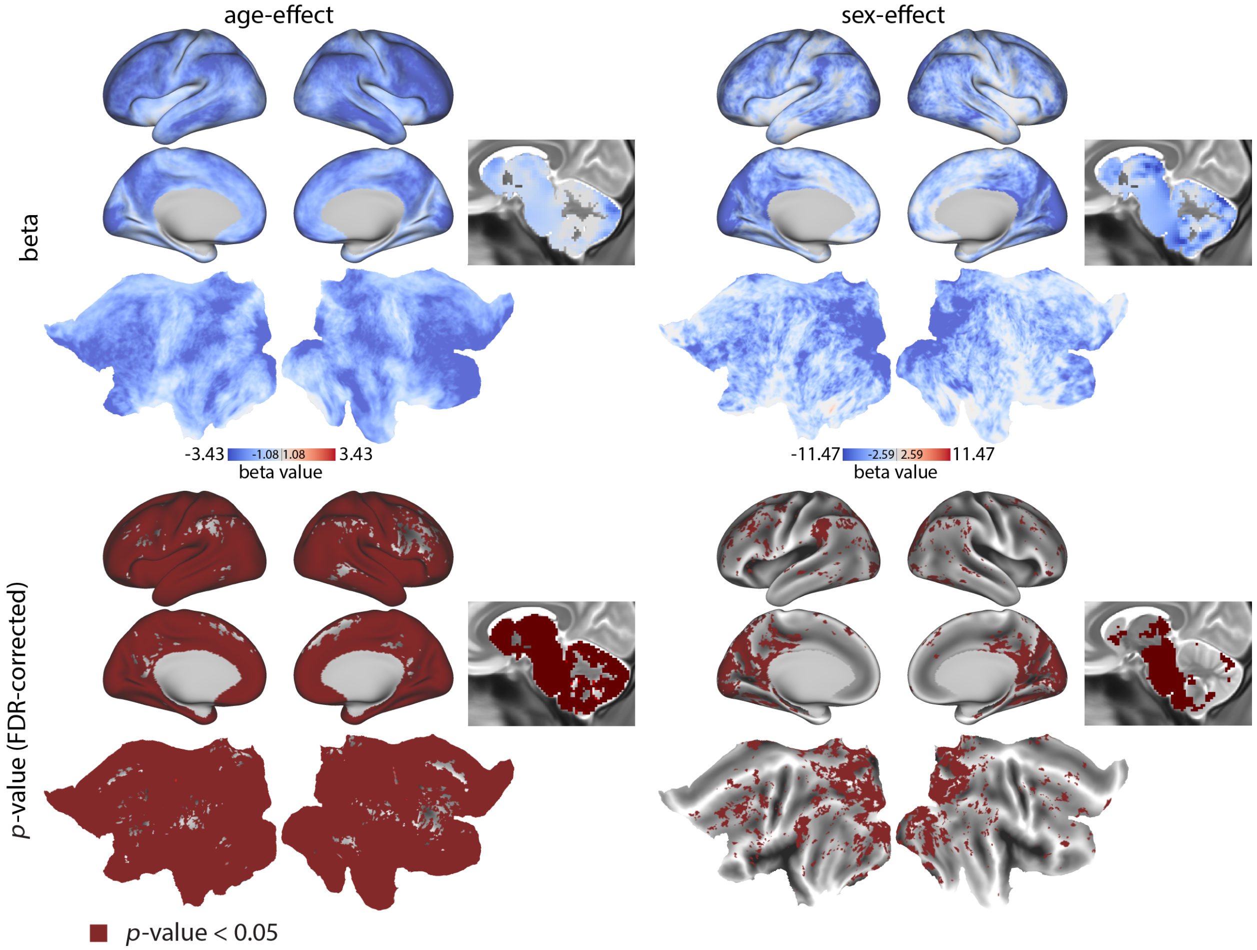

Figure S12. **Age and sex-effects of cerebral blood perfusion in the HCP-D dataset |** Top: The general linear model (GLM) coefficients for age and biological sex (male $= 1$, female $= 0$) are shown on the cortical fsLR surface maps and MNI152 T2-weighted volume space. Bottom: Areas with Bonferroni-corrected p-values below 0.05 are marked in red.
