# Supplementary material for: Mapping cerebral blood perfusion and its links to multi-scale brain organization across the human lifespan
Source: PLoS Biol. 2025 Jul 29;23(7):e3003277. doi: 10.1371/journal.pbio.3003277 (PMC12324687; doi:10.1371/journal.pbio.3003277)
Supplement: S14 Fig — (a) Each dot corresponds to a participant’s mean brain blood perfusion level in the HCP-D cohort (male: blue, female: red). A separate GAMLSS model is fitted for each sex-group to capture age-related changes in grayordinates’ perfusion during development. (b) Parcel-wise trajectories of cerebral blood perfusion across age are modeled using GAMLSS in each of the 400 Schaefer parcels. Each panel displays the fitted trajectories for male and female participants. (c) Model fit values are shown on lateral and medial views of the inflated and 2D flat cortical surfaces (fsLR). Higher values indicate better fits. (d) Comparison of mean model fit values across seven canonical intrinsic functional networks introduced by Yeo et al. [124] shows that transmodal regions exhibit higher model fits than unimodal regions. Additionally, model fits are higher in males than in females. (PDF) [file pbio.3003277.s014.pdf]

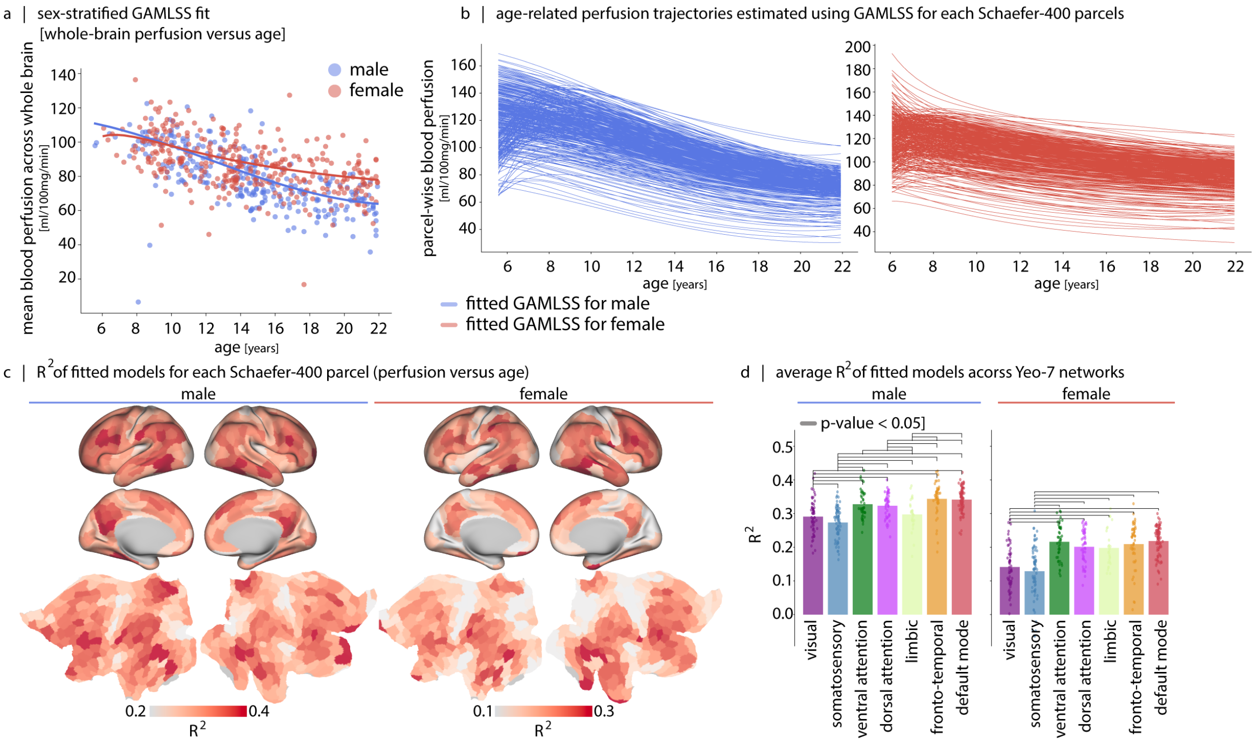

Figure S14. **GAMLSS trajectories to model blood perfusion (versus age) in development |** (a) Each dot corresponds to a participant's mean brain blood perfusion level in the HCP-D cohort (male: blue, female: red). A separate GAMLSS model is fitted for each sex-group to capture age-related changes in grayordinates' perfusion during development. (b) Parcel-wise trajectories of cerebral blood perfusion across age are modeled using GAMLSS in each of the 400 Schaefer parcels. Each panel displays the fitted trajectories for male and female participants. (c) Model fit values are shown on lateral and medial views of the inflated and 2D flat cortical surfaces (fsLR). Higher values indicate better fits. (d) Comparison of mean model fit values across seven canonical intrinsic functional networks introduced by Yeo et al. [1] shows that transmodal regions exhibit higher model fits than unimodal regions. Additionally, model fits are higher in males than in females.

References

1. Yeo BT, Krienen FM, Sepulcre J, Sabuncu MR, Lashkari D, Hollinshead M, et al. The organization of the human cerebral cortex estimated by intrinsic functional connectivity. *Journal of neurophysiology*. 2011.
