# Supplementary material for: Mapping cerebral blood perfusion and its links to multi-scale brain organization across the human lifespan
Source: PLoS Biol. 2025 Jul 29;23(7):e3003277. doi: 10.1371/journal.pbio.3003277 (PMC12324687; doi:10.1371/journal.pbio.3003277)
Supplement: S15 Fig — Fitted GAMLSS trajectories of cerebral perfusion are shown for each of the 400 Schaefer parcels, grouped by seven canonical intrinsic functional networks introduced by Yeo et al. [124]. (PDF) [file pbio.3003277.s015.pdf]

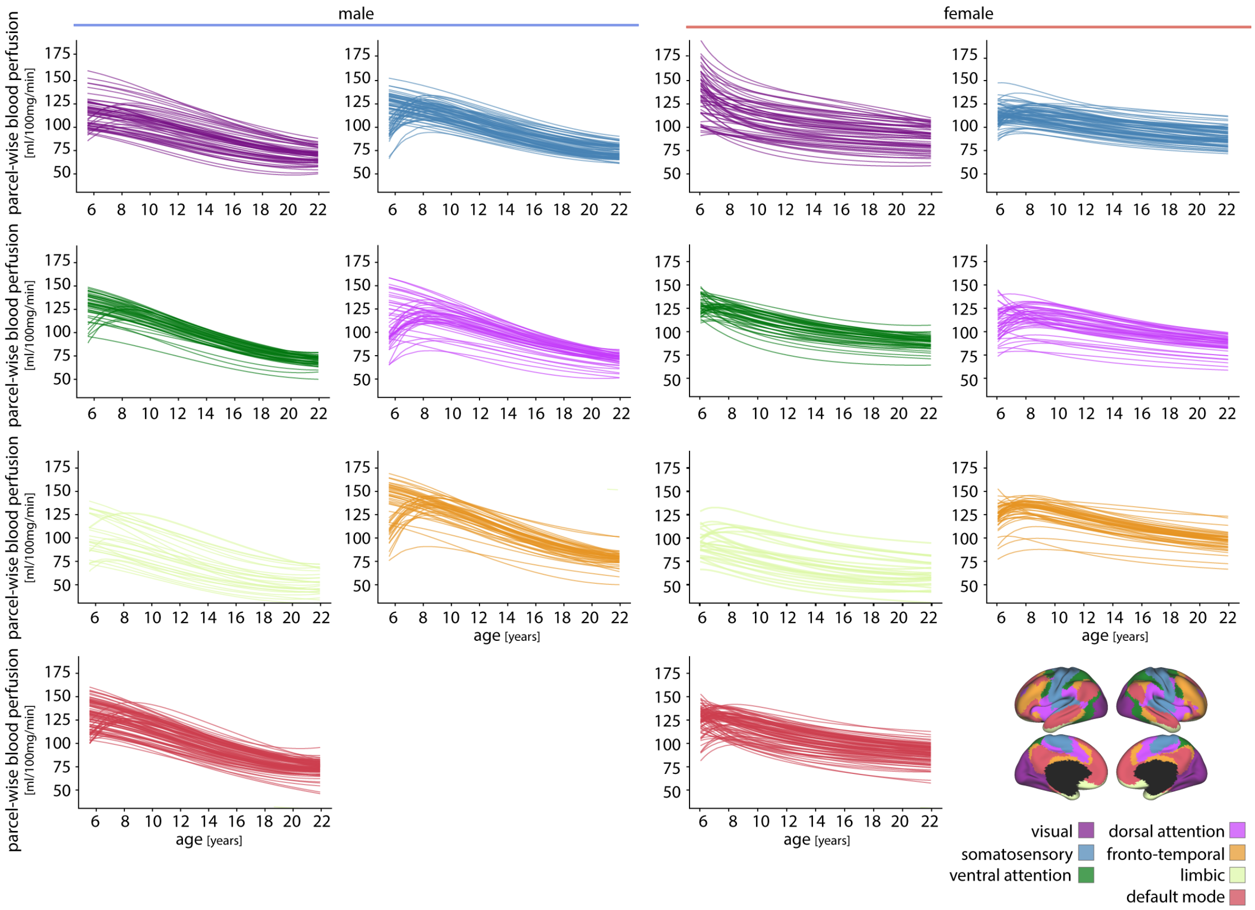

Figure S15. **Sex-stratified trajectories of perfusion during development |** Fitted GAMLSS trajectories of cerebral perfusion are shown for each of the 400 Schaefer parcels, grouped by seven canonical intrinsic functional networks introduced by Yeo et al. [1].

References

1. Yeo BT, Krienen FM, Sepulcre J, Sabuncu MR, Lashkari D, Hollinshead M, et al. The organization of the human cerebral cortex estimated by intrinsic functional connectivity. *Journal of neurophysiology*. 2011.
