# Supplementary material for: Mapping cerebral blood perfusion and its links to multi-scale brain organization across the human lifespan
Source: PLoS Biol. 2025 Jul 29;23(7):e3003277. doi: 10.1371/journal.pbio.3003277 (PMC12324687; doi:10.1371/journal.pbio.3003277)
Supplement: S17 Fig — (PDF) [file pbio.3003277.s017.pdf]

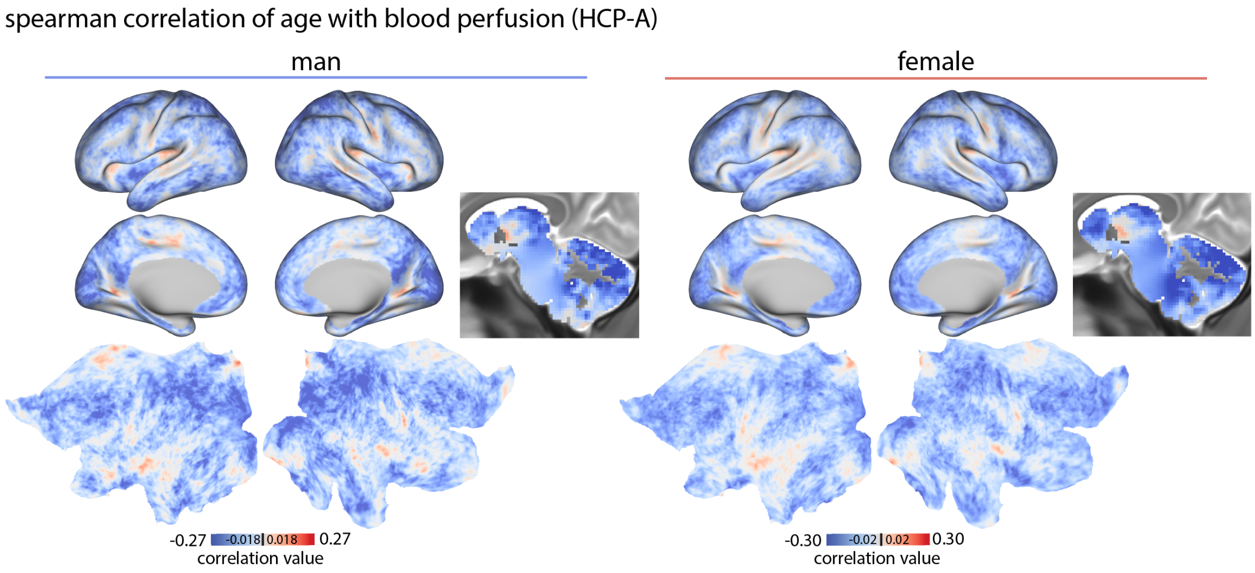

Figure S17. **Spearman correlation between cerebral blood perfusion and age in the HCP-A dataset**
