# Supplementary material for: Mapping cerebral blood perfusion and its links to multi-scale brain organization across the human lifespan
Source: PLoS Biol. 2025 Jul 29;23(7):e3003277. doi: 10.1371/journal.pbio.3003277 (PMC12324687; doi:10.1371/journal.pbio.3003277)
Supplement: S18 Fig — (a) Each dot corresponds to a participant’s mean brain blood perfusion level in the HCP-A cohort (male: blue, female: red). A separate GAMLSS model is fitted for each sex-group to capture age-related changes in grayordinates’ perfusion during aging. (b) Parcel-wise trajectories of cerebral blood perfusion across age are modeled using GAMLSS in each of the 400 Schaefer parcels. Model fit values are shown on lateral and medial views of the inflated and 2D flat cortical surfaces (fsLR). Higher values indicate better fits. (PDF) [file pbio.3003277.s018.pdf]

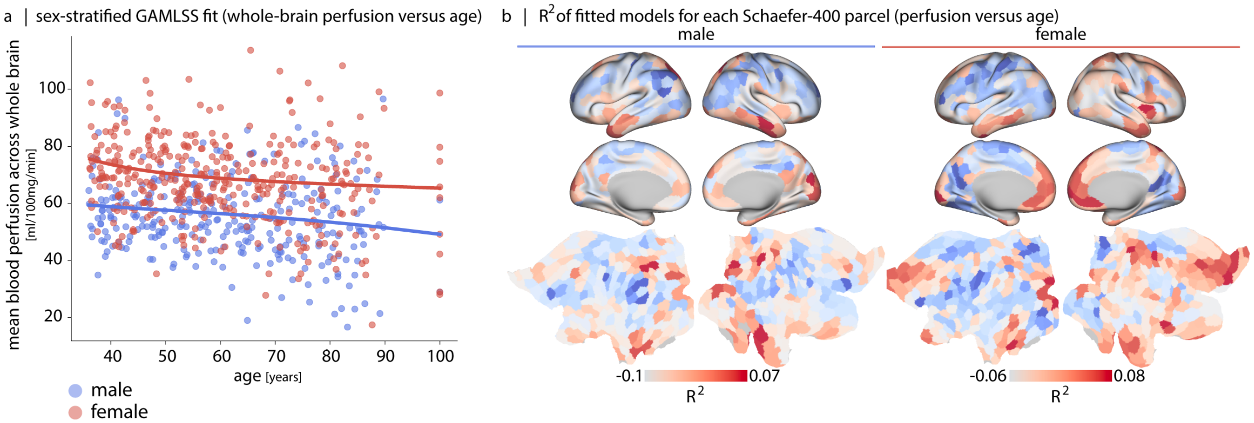

Figure S18. **GAMLSS trajectories to model blood perfusion (versus age) in aging |** (a) Each dot corresponds to a participant's mean brain blood perfusion level in the HCP-A cohort (male: blue, female: red). A separate GAMLSS model is fitted for each sex-group to capture age-related changes in grayordinates' perfusion during aging. (b) Parcel-wise trajectories of cerebral blood perfusion across age are modeled using GAMLSS in each of the 400 Schaefer parcels. Model fit values are shown on lateral and medial views of the inflated and 2D flat cortical surfaces (fsLR). Higher values indicate better fits.
