# Supplementary material for: Mapping cerebral blood perfusion and its links to multi-scale brain organization across the human lifespan
Source: PLoS Biol. 2025 Jul 29;23(7):e3003277. doi: 10.1371/journal.pbio.3003277 (PMC12324687; doi:10.1371/journal.pbio.3003277)
Supplement: S19 Fig — (a) To generate a representative arterial transit time (ATT) map, the vertex/voxel-wise ATT map of participants are z-scored and then combined into a single data matrix. Principal component analysis (PCA) is applied to derive the first principal component (PC1; explaining 22.9% of the variance). The first PC score map is shown on lateral and medial views of the inflated and 2D flat cortical surfaces (fsLR). The volumetric part of the map is shown on the sagittal view of the T2-weighted group-average template (MNI152). Here positive values mark regions with late blood arrival times. The second principal component explains 4.07% of the variance in the data. (b) Loadings of the first principal component are shown per participant (male: blue, female: red). (PDF) [file pbio.3003277.s019.pdf]

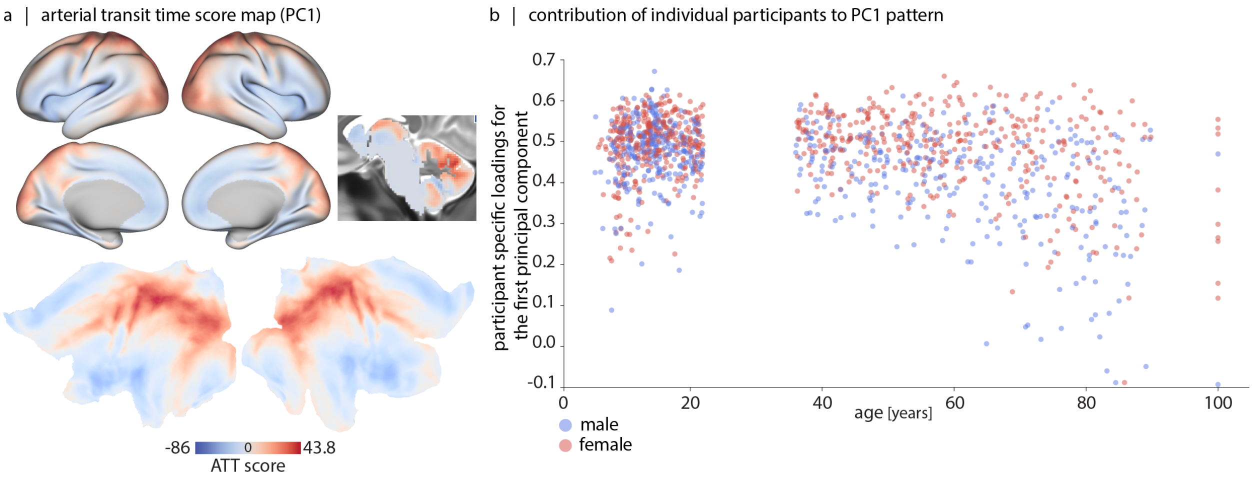

Figure S19. **First principal component of arterial transit time across HCP-D and HCP-A participants |** (a) To generate a representative arterial transit time (ATT) map, the vertex/voxel-wise ATT map of participants are z-scored and then combined into a single data matrix. Principal component analysis (PCA) is applied to derive the first principal component (PC1; explaining 22.9% of the variance). The first PC score map is shown on lateral and medial views of the inflated and 2D flat cortical surfaces (fsLR). The volumetric part of the map is shown on the sagittal view of the T2-weighted group-average template (MNI152). Here positive values mark regions with late blood arrival times. The second principal component explains 4.07% of the variance in the data. (b) Loadings of the first principal component are shown per participant (male: blue, female: red).
