# Supplementary material for: Mapping cerebral blood perfusion and its links to multi-scale brain organization across the human lifespan
Source: PLoS Biol. 2025 Jul 29;23(7):e3003277. doi: 10.1371/journal.pbio.3003277 (PMC12324687; doi:10.1371/journal.pbio.3003277)
Supplement: S20 Fig — Relationship between the raw biomarkers used in the PLS analysis (y-axis) and participants’ age (x-axis) (male: blue, female: red). (PDF) [file pbio.3003277.s020.pdf]

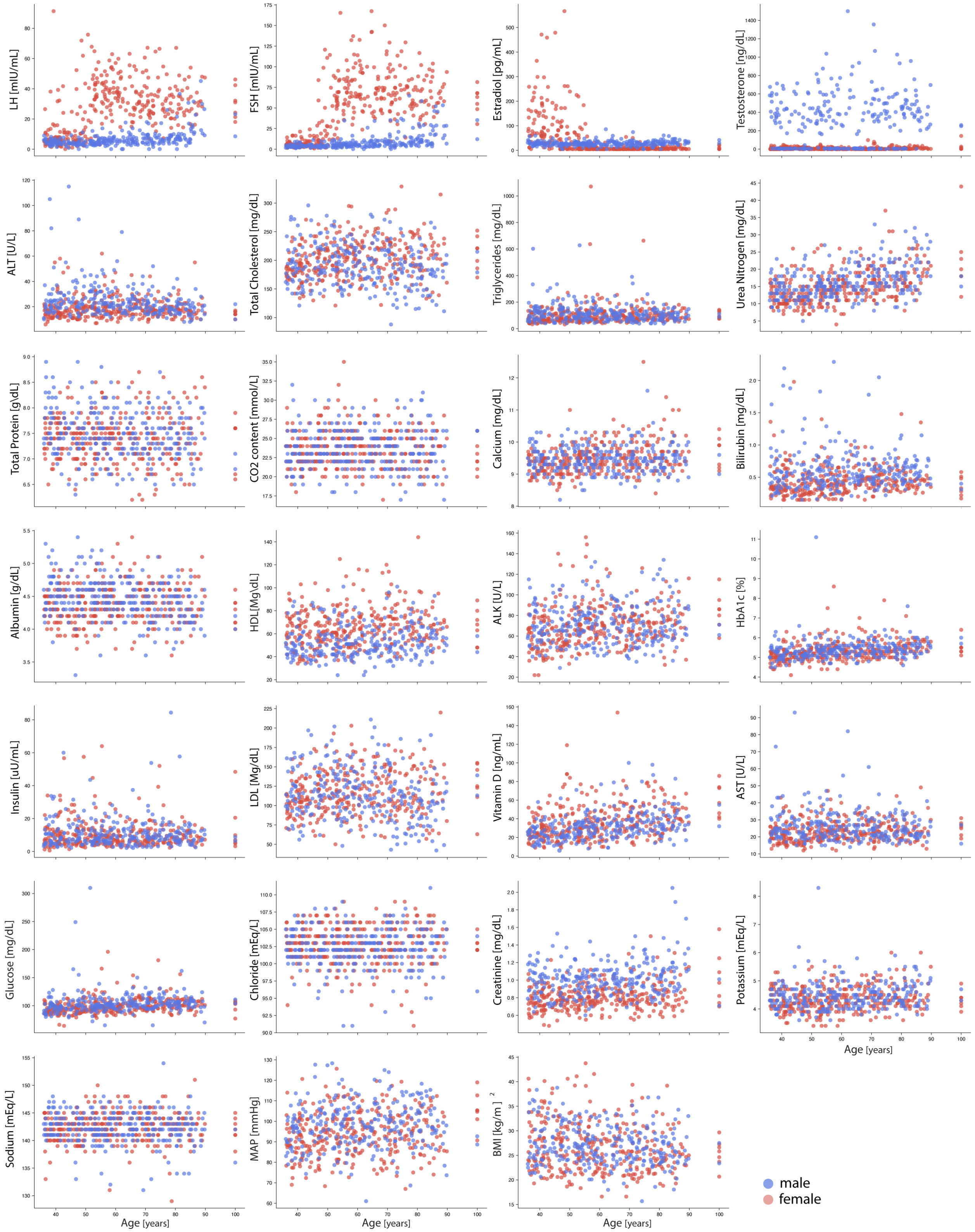

Figure S20. **Biomarkers versus age |** Relationship between the raw biomarkers used in the PLS analysis (y-axis) and participants' age (x-axis) (male: blue, female: red).
