# Supplementary material for: Mapping cerebral blood perfusion and its links to multi-scale brain organization across the human lifespan
Source: PLoS Biol. 2025 Jul 29;23(7):e3003277. doi: 10.1371/journal.pbio.3003277 (PMC12324687; doi:10.1371/journal.pbio.3003277)
Supplement: S21 Fig — PLS bootstrapping is used to assess the robustness and stability of PLS weights [291,292]. (PDF) [file pbio.3003277.s021.pdf]

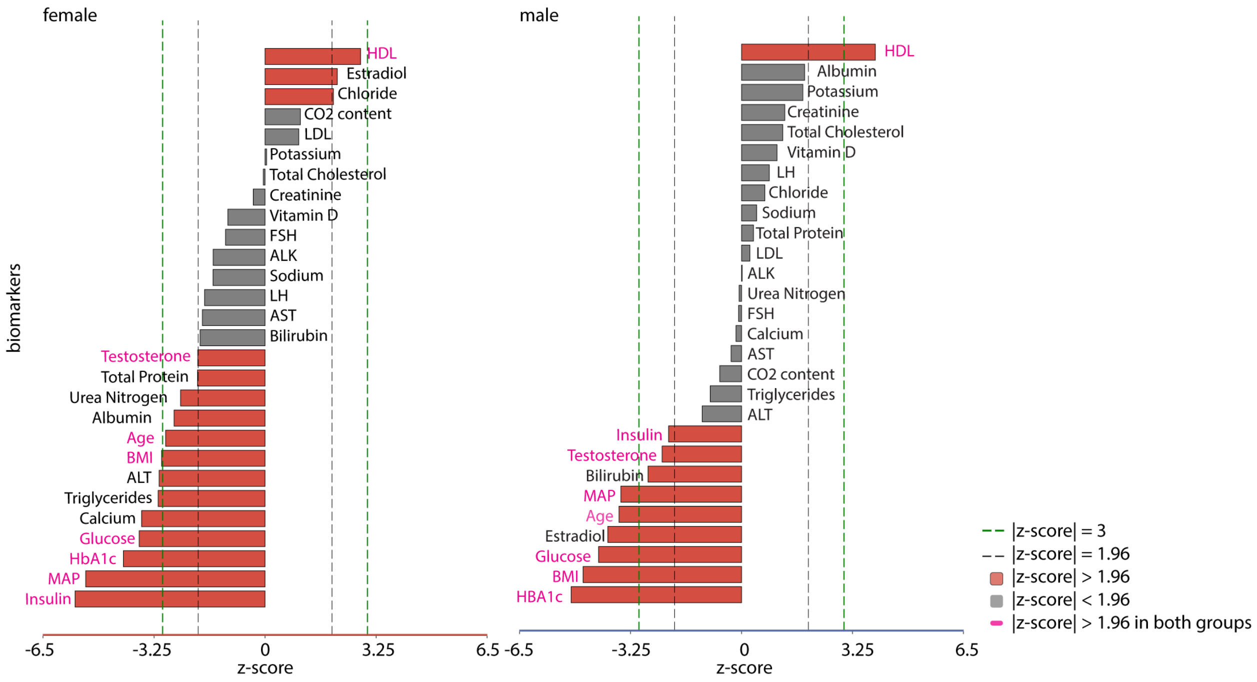

Figure S21. **The z-scored weight distribution of biomarkers |** PLS bootstrapping is used to assess the robustness and stability of PLS weights [1, 2].

References

1. Morgan SE, Seidlitz J, Whitaker KJ, Romero-Garcia R, Clifton NE, Scarpazza C, et al. Cortical patterning of abnormal morphometric similarity in psychosis is associated with brain expression of schizophrenia-related genes. *Proceedings of the National Academy of Science*. 2019;116(19):9604–9609.

2. Zhukovsky P, Wainberg M, Milic M, Tripathy SJ, Mulsant BH, Felsky D, et al. Multiscale neural signatures of major depressive, anxiety, and stress-related disorders. *Proceedings of the National Academy of Science*. 2022;119(23):e2204433119.
