# Supplementary material for: Mapping cerebral blood perfusion and its links to multi-scale brain organization across the human lifespan
Source: PLoS Biol. 2025 Jul 29;23(7):e3003277. doi: 10.1371/journal.pbio.3003277 (PMC12324687; doi:10.1371/journal.pbio.3003277)
Supplement: S22 Fig — (a) Using a GLM model we regress out the linear effect of age and sex from both the biomarker data and the vertex/voxel-wise perfusion data, we parcellate the perfusion maps using Schaefer-400 atlas and relate the biomarkers (excluding age and sex) to blood perfusion maps of participants using the partial least squares (PLS) analysis. The first latent variable accounts for 94.30% of the covariance between cortical blood perfusion and biomarker profiles of HCP-A participants (p=9.99×10^−4^, 1000 repetitions). (b) The bar plot visualizes the contribution (effect size) of individual biomarkers to the first latent variable. The significance of each biomarker’s contribution to the overall pattern is assessed by bootstrap resampling (1000 repetitions). (c) The brain loadings of the first latent variable are shown on the inflated and 2D flat cortical surfaces (fsLR). (PDF) [file pbio.3003277.s022.pdf]

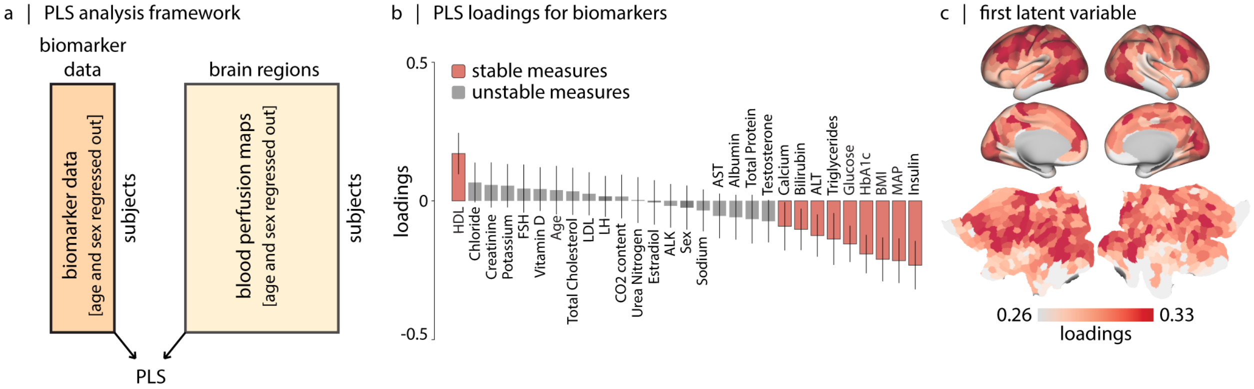

Figure S22. **Relating blood perfusion and biomarkers after regressing out the linear effect of age and sex |** (a) Using a GLM model we regress out the linear effect of age and sex from both the biomarker data and the vertex/voxel-wise perfusion data, we parcellate the perfusion maps using Schaefer-400 atlas and relate the biomarkers (excluding age and sex) to blood perfusion maps of participants using the partial least squares (PLS) analysis. The first latent variable accounts for 94.30% of the covariance between cortical blood perfusion and biomarker profiles of HCP-A participants ($p = 9.99 \times 10^{-4}$, 1 000 repetitions). (b) The bar plot visualizes the contribution (effect size) of individual biomarkers to the first latent variable. The significance of each biomarker's contribution to the overall pattern is assessed by bootstrap resampling (1 000 repetitions). (c) The brain loadings of the first latent variable are shown on the inflated and 2D flat cortical surfaces (fsLR).
