# Supplementary material for: Mapping cerebral blood perfusion and its links to multi-scale brain organization across the human lifespan
Source: PLoS Biol. 2025 Jul 29;23(7):e3003277. doi: 10.1371/journal.pbio.3003277 (PMC12324687; doi:10.1371/journal.pbio.3003277)
Supplement: S23 Fig — The S4 Tian parcels are used to parcellate the gradient values in subcortex [87]. See S3 Table for full parcel names. S = superior; A = anterior; P = posterior; I = inferior; R = right; L = left. (PDF) [file pbio.3003277.s023.pdf]

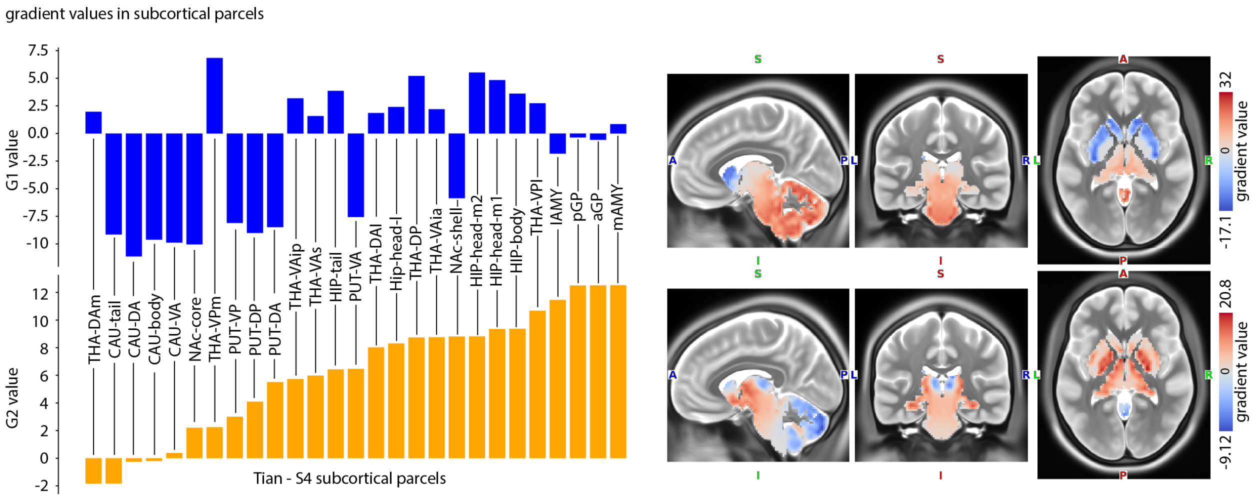

Figure S23. **Cerebral blood perfusion covariance matrix gradients in subcortex |** The S4 Tian parcels are used to parcellate the gradient values in subcortex [1]. See S3 Table for full parcel names. S = superior; A = anterior; P = posterior; I = inferior; R = right; L = left.

References

1. Tian Y, Margulies DS, Breakspear M, Zalesky A. Topographic organization of the human subcortex unveiled with functional connectivity gradients. *Nature Neuroscience*. 2020;23(11):1421–1432.
