# Supplementary material for: Mapping cerebral blood perfusion and its links to multi-scale brain organization across the human lifespan
Source: PLoS Biol. 2025 Jul 29;23(7):e3003277. doi: 10.1371/journal.pbio.3003277 (PMC12324687; doi:10.1371/journal.pbio.3003277)
Supplement: S2 Table — Parcels are defined based on the multi-modal Glasser parcellation of cerebral cortex, which includes 180 parcels in each hemisphere [86]. For each parcel, we calculate the mean perfusion scores across both hemispheres. This approach ensures that we have a single value per parcel, rather than separate values for the left and right hemispheres. Fig 2A shows a detailed map with areal borders overlaid on the perfusion map. Full name of parcels is provided in S1 Table. (PDF) [file pbio.3003277.s025.pdf]

**Mean blood perfusion score per cortical parcel**									
A1	LBelt	RSC	a9-46v	MBelt	p47r	p10p	POS2	8C	a10p
57.77	46.91	41.32	40.59	40.52	39.29	37.04	34.56	34.11	33.84
PBelt	p9-46v	3b	V1	IFSa	3a	IFSp	PEF	23d	a47r
33.69	33.69	33.12	32.84	32.64	32.50	31.46	30.35	30.13	29.61
RI	9-46d	52	55b	23c	IFJa	IP1	FEF	PFm	IFJp
29.57	29.35	29.35	29.28	29.12	28.63	28.50	27.92	27.53	27.50
31a	V3A	i6-8	46	PGs	s6-8	8Av	45	V7	A4
27.36	27.36	26.65	26.62	26.37	26.26	26.13	26.12	26.10	25.96
LIPd	43	9a	IP2	8Ad	44	FOP5	V3B	LIPv	6r
25.71	25.29	25.17	24.63	24.41	24.33	23.65	23.37	23.33	23.31
V3	31pd	10d	47m	9p	31pv	V2	LO1	6v	V3CD
23.24	23.13	22.96	22.67	22.49	22.45	21.68	21.26	21.14	21.06
6ma	PF	10r	MIP	33pr	IPS1	V4	d23ab	p32	V6
21.05	20.84	20.61	20.19	19.99	19.52	19.37	19.25	19.22	19.03
SCEF	7Pm	PSL	47l	p32pr	FOP4	8BM	6a	8BL	TA2
18.69	18.68	18.68	18.18	18.03	17.93	17.80	17.28	16.49	16.46
7Pl	MST	IP0	PGp	5mv	1	7m	DVT	AIP	a32pr
16.38	16.23	16.14	16.11	15.75	15.46	14.70	14.46	14.44	14.34
SFL	MT	AVI	4	STSvp	FOP1	LO3	OP2-3	PHT	STV
14.18	13.75	13.50	13.48	13.44	13.41	13.37	13.34	13.32	13.29
PGi	p24pr	LO2	24dv	V6A	TPOJ3	v23ab	24dd	PCV	TPOJ1
13.12	12.93	12.81	12.75	12.51	12.42	12.34	11.98	11.70	11.54
13l	FOP3	11l	d32	TPOJ2	V4t	A5	a24pr	PFop	TE1p
11.11	11.08	11.00	11.00	10.97	10.79	10.78	10.67	10.63	10.44
6mp	2	POS1	PFcm	VIP	7Am	6d	5m	PFt	9m
10.34	10.20	10.09	9.83	9.69	9.67	9.46	8.94	7.89	7.82
OP4	PIT	a24	STSdp	7PC	7AL	MI	FST	STSda	s32
7.42	6.76	6.53	6.50	6.25	5.34	4.54	4.45	4.38	4.11
STSva	Ig	5L	47s	OP1	pOFC	VMV2	p24	PI	V8
3.46	2.69	2.09	1.63	1.50	0.71	0.63	0.30	0.13	0.03
ProS	VMV3	VMV1	TE1m	PH	FOP2	25	10pp	PHA1	PreS
-0.56	-1.64	-2.05	-2.06	-2.29	-4.18	-6.81	-6.90	-8.34	-9.02
STGa	PoI1	PHA2	FFC	TE1a	PHA3	VVC	TE2p	10v	PoI2
-9.22	-9.30	-10.01	-11.20	-13.04	-13.76	-15.56	-15.80	-17.54	-17.75
OFC	AAIC	TGd	EC	TGv	H	Pir	TF	PeEc	TE2a
-18.97	-19.29	-20.15	-28.92	-29.04	-30.33	-32.98	-39.96	-40.39	-42.09

TABLE S2. **Mean blood perfusion score per cortical parcel |** Parcels are defined based on the multi-modal Glasser parcellation of cerebral cortex, which includes 180 parcels in each hemisphere [1]. For each parcel, we calculate the mean perfusion scores across both hemispheres. This approach ensures that we have a single value per parcel, rather than separate values for the left and right hemispheres. Fig 2A shows a detailed map with areal borders overlaid on the perfusion map. Full name of parcels is provided in S1 Table.

References

1. Glasser MF, Coalson TS, Robinson EC, Hacker CD, Harwell J, Yacoub E, et al. A multi-modal parcellation of human cerebral cortex. *Nature*. 2016;536(7615):171–178.
