# Supplementary material for: Mapping cerebral blood perfusion and its links to multi-scale brain organization across the human lifespan
Source: PLoS Biol. 2025 Jul 29;23(7):e3003277. doi: 10.1371/journal.pbio.3003277 (PMC12324687; doi:10.1371/journal.pbio.3003277)
Supplement: S3 Table — (PDF) [file pbio.3003277.s026.pdf]

Name	Abbreviation	Name	Abbreviation
**Anterior hippocampus**		**Posterior hippocampus**	
Hippocampus head, medial division - Subdivision 1	HIP-head-m1	Hippocampus body	HIP-body
Hippocampus head, medial division - Subdivision 2	HIP-head-m2	Hippocampus tail	HIP-tail
Hippocampus head, lateral division	HIP-head-l		
**Amygdala**			
Lateral amygdala	LAMY	Medial amygdala	mAMY
**Anterior caudate**		**Posterior caudate**	
Dorsoanterior caudate	CAU-DA	Caudate tail	CAU-tail
Ventroanterior caudate	CAU-VA	Caudate body	CAU-body
**Nucleus accumbens**			
Nucleus accumbens, shell	NAc-shell	Nucleus accumbens, core	NAc-core
**Anterior putamen**		**Posterior putamen**	
Dorsoanterior putamen	PUT-DA	Dorsoposterior putamen	PUT-DP
Ventroanterior putamen	PUT-VA	Ventroposterior putamen	PUT-VP
**Globus pallidus**			
Anterior globus pallidus	aGP	Posterior globus pallidus	pGP
**Anterior thalamus**		**Posterior thalamus**	
Lateral dorsoanterior thalamus	THA-DAl	Dorsoposterior thalamus	THA-DP
Medial dorsoanterior thalamus	THA-DAm	Medial ventroposterior thalamus	THA-VPm
Superior ventroanterior thalamus	THA-VAs	Lateral ventroposterior thalamus	THA-VPl
Inferior ventroanterior thalamus - Anterior division	THA-VAia		
Inferior ventroanterior thalamus - Posterior division	THA-VAip		

TABLE S3: **Nomenclature for Tian-S4 subcortical functional parcellation [1].**

References

1. Tian Y, Margulies DS, Breakspear M, Zalesky A. Topographic organization of the human subcortex unveiled with functional connectivity gradients. *Nature Neuroscience*. 2020;23(11):1421–1432.
