# Supplementary material for: Mapping cerebral blood perfusion and its links to multi-scale brain organization across the human lifespan
Source: PLoS Biol. 2025 Jul 29;23(7):e3003277. doi: 10.1371/journal.pbio.3003277 (PMC12324687; doi:10.1371/journal.pbio.3003277)
Supplement: S6 Table — Values in parentheses (under #Participants) indicate number of females. (PDF) [file pbio.3003277.s029.pdf]

Receptor/ transporter	Neurotransmitter	Tracer	#Participants	Age (years)	References
5-HT_{1A}	serotonin	[^{11}C]WAY-100635	35 (17)	26.3 ± 5.2	Beliveau et al., 2017 [1]
5-HT_{1B}	serotonin	[^{11}C]P943	65 (16)	33.7 ± 9.7	Gallezot et al., 2010 [2-8]
5-HT_{2A}	serotonin	[^{11}C]Cimbi-36	29 (14)	22.6 ± 2.7	Beliveau et al., 2017 [1]
5-HT_4	serotonin	[^{11}C]SB207145	59 (18)	25.9 ± 5.3	Beliveau et al., 2017 [1]
5-HT_6	serotonin	[^{11}C]GSK215083	30 (0)	36.6 ± 9.0	Radhakrishnan et al., 2018 [9, 10]
NMDA	glutamate	[^{18}F]GE-179	29 (8)	40.9 ± 12.7	Galovic et al., 2021 [11-13]
$GABA_{A/BZ}$	GABA	[^{11}C]flumazenil	16 (9)	26.6 ± 8	Nørgaard et al., 2021 [14]

TABLE S6. **Neurotransmitter receptors |** Values in parentheses (under #Participants) indicate number of females.

References

1. Beliveau V, Ganz M, Feng L, Ozenne B, Højgaard L, Fisher PM, et al. A high-resolution in vivo atlas of the human brain's serotonin system. *Journal of Neuroscience*. 2017;37(1):120–128.
2. Gallezot JD, Nabulsi N, Neumeister A, Planeta-Wilson B, Williams WA, Singhal T, et al. Kinetic modeling of the serotonin 5-HT1B receptor radioligand [11C] P943 in humans. *Journal of Cerebral Blood Flow & Metabolism*. 2010;30(1):196–210.
3. Murrough JW, Henry S, Hu J, Gallezot JD, Planeta-Wilson B, Neumaier JF, et al. Reduced ventral striatal/ventral pallidal serotonin 1b receptor binding potential in major depressive disorder. *Psychopharmacology*. 2011;213:547–553.
4. Murrough JW, Czermak C, Henry S, Nabulsi N, Gallezot JD, Gueorguieva R, et al. The effect of early trauma exposure on serotonin type 1B receptor expression revealed by reduced selective radioligand binding. *Archives of general psychiatry*. 2011;68(9):892–900.
5. Matuskey D, Bhagwagar Z, Planeta B, Pittman B, Gallezot JD, Chen J, et al. Reductions in brain 5-HT1B receptor availability in primarily cocaine-dependent humans. *Biological psychiatry*. 2014;76(10):816–822.
6. Pittenger C, Adams Jr TG, Gallezot JD, Crowley MJ, Nabulsi N, Ropchan J, et al. OCD is associated with an altered association between sensorimotor gating and cortical and subcortical 5-HT1b receptor binding. *Journal of affective disorders*. 2016;196:87–96.
7. Saricicek A, Chen J, Planeta B, Ruf B, Subramanyam K, Maloney K, et al. Test–retest reliability of the novel 5-ht 1b receptor pet radioligand [11 c] p943. *European journal of nuclear medicine and molecular imaging*. 2015;42:468–477.
8. Baldassarri SR, Park E, Finnema SJ, Planeta B, Nabulsi N, Najafzadeh S, et al. Inverse changes in raphe and cortical 5-HT1B receptor availability after acute tryptophan depletion in healthy human subjects. *Synapse*. 2020;74(10):e22159.
9. Radhakrishnan R, Nabulsi N, Gaiser E, Gallezot JD, Henry S, Planeta B, et al. Age-related change in 5-HT6 receptor availability in healthy male volunteers measured with 11C-GSK215083 PET. *Journal of Nuclear Medicine*. 2018;59(9):1445–1450.
10. Radhakrishnan R, Matuskey D, Nabulsi N, Gaiser E, Gallezot JD, Henry S, et al. In vivo 5-HT6 and 5-HT2A receptor availability in antipsychotic treated schizophrenia patients vs. unmedicated healthy humans measured with [11C] GSK215083 PET. *Psychiatry Research: Neuroimaging*. 2020;295:111007.
11. Galovic M, Al-Diwani A, Vivekananda U, Torrealdea F, Erlandsson K, Fryer TD, et al. In vivo NMDA receptor function in people with NMDA receptor antibody encephalitis. *medRxiv*. 2021; p. 2021–12.
12. Galovic M, Erlandsson K, Fryer TD, Hong YT, Manavaki R, Sari H, et al. Validation of a combined image derived input function and venous sampling approach for the quantification of [18F] GE-179 PET binding in the brain. *Neuroimage*. 2021;237:118194.
13. McGinnity CJ, Hammers A, Barros DAR, Luthra SK, Jones PA, Trigg W, et al. Initial evaluation of 18F-GE-179, a putative PET tracer for activated N-methyl D-aspartate receptors. *Journal of Nuclear Medicine*. 2014;55(3):423–430.
14. Nørgaard M, Beliveau V, Ganz M, Svarer C, Pinborg LH, Keller SH, et al. A high-resolution in vivo atlas of the human brain's benzodiazepine binding site of GABAA receptors. *NeuroImage*. 2021;232:117878.
