# Supplementary material for: Mapping cerebral blood perfusion and its links to multi-scale brain organization across the human lifespan
Source: PLoS Biol. 2025 Jul 29;23(7):e3003277. doi: 10.1371/journal.pbio.3003277 (PMC12324687; doi:10.1371/journal.pbio.3003277)
Supplement: S7 Table — The t-statistics and p-values per each age-bin are shown. “#” denotes the number of participants. (PDF) [file pbio.3003277.s030.pdf]

**Age Bin**	**#Male**	**#Female**	t**-statistic**	p**-value**
<9	23	34	-0.8088	4.2529e-01
9-10	20	39	-0.5507	5.8469e-01
10-11	24	22	-0.9784	3.3330e-01
11-12	19	16	-0.4404	6.6357e-01
12-13	20	24	0.5778	5.6653e-01
13-15	64	62	-2.3889	1.8474e-02
15-17	42	46	-4.7678	7.5291e-06
17-19	27	36	-4.3597	5.2314e-05
19-21	33	36	-4.3922	4.1910e-05
21-23	18	22	-4.0244	2.6888e-04
29-35	0	0	–	–
35-47	67	98	-6.7027	3.9130e-10
47-57	65	88	-6.7457	3.0873e-10
57-67	58	66	-5.4027	3.5573e-07
67-77	47	61	-3.6007	4.8620e-04
>77	60	68	-3.9912	1.1106e-04

TABLE S7. **Comparison of male and female cerebral blood perfusion values in different age bins |** The t-statistics and p-values per each age-bin are shown. "#" denotes the number of participants.
