# Supplementary material for: Mapping cerebral blood perfusion and its links to multi-scale brain organization across the human lifespan
Source: PLoS Biol. 2025 Jul 29;23(7):e3003277. doi: 10.1371/journal.pbio.3003277 (PMC12324687; doi:10.1371/journal.pbio.3003277)
Supplement: S8 Table — List of 124 used Neurosynth terms in this study. (PDF) [file pbio.3003277.s031.pdf]

| Neurosynth terms | | | |
|---|---|---|---|
| action | adaptation | addiction | anticipation |
| anxiety | arousal | association | attention |
| autobiographical_memory | balance | belief | categorization |
| cognitive_control | communication | competition | concept |
| consciousness | consolidation | context | coordination |
| decision | decision_making | detection | discrimination |
| distraction | eating | efficiency | effort |
| emotion | emotion_regulation | empathy | encoding |
| episodic_memory | expectancy | expertise | extinction |
| face_recognition | facial_expression | familiarity | fear |
| fixation | focus | gaze | goal |
| hyperactivity | imagery | impulsivity | induction |
| inference | inhibition | insight | integration |
| intelligence | intention | interference | judgment |
| knowledge | language | language_comprehension | learning |
| listening | localization | loss | maintenance |
| manipulation | meaning | memory | memory_retrieval |
| mental_imagery | monitoring | mood | morphology |
| motor_control | movement | multisensory | naming |
| navigation | object_recognition | pain | perception |
| planning | priming | psychosis | reading |
| reasoning | recall | recognition | rehearsal |
| reinforcement_learning | response_inhibition | response_selection | retention |
| retrieval | reward_anticipation | rhythm | risk |
| rule | salience | search | selective_attention |
| semantic_memory | sentence_comprehension | skill | sleep |
| social_cognition | spatial_attention | speech_perception | speech_production |
| strategy | strength | stress | sustained_attention |
| task_difficulty | thought | timing | uncertainty |
| updating | utility | valence | verbal_fluency |
| visual_attention | visual_perception | word_recognition | working_memory |

TABLE S8: **Neurosynth terms |** List of 124 used Neurosynth terms in this study.
