# Supplementary material for: Mapping cerebral blood perfusion and its links to multi-scale brain organization across the human lifespan
Source: PLoS Biol. 2025 Jul 29;23(7):e3003277. doi: 10.1371/journal.pbio.3003277 (PMC12324687; doi:10.1371/journal.pbio.3003277)
Supplement: Attachment — Submitted filename: response_to_revieweres.pdf [file pbio.3003277.s032.pdf]

Dear Dr. Smith and Reviewers,

Thank you for the constructive feedback on our first submission and for the opportunity to improve the manuscript. Following your comments and suggestions, we have substantially revised the manuscript. Reviewer comments are in **bold font** and our responses are in regular font. Major changes in the manuscript include:

- Added the white-matter blood perfusion trajectory to more comprehensively characterize the age-related changes.
- Expanded the discussion section to interpret the coupling between plasma biosignatures and brain blood perfusion, providing greater contextual insight for the findings.
- Included additional statistical tests to assess age and sex interaction on blood perfusion during development.
- Added more validation for the results coming from the PLS analysis.
- Modeled the non-linear age effects across development and aging using GAMLSS.
- More comprehensive analysis of the relationship between cortical layer architecture and perfusion patterns.
- Clarification of methodological details, particularly regarding the use of parcellations.

Yours sincerely,

Bratislav Misic, PhD

Montreal Neurological Institute

Reviewer #1,
Danny JJ Wang:

This is a revision, the authors have been responsive to previous review, their methods are quite rigorous. I only have a few minor comments:

1) Have you tried to look at the differences in the distribution and magnitude of perfusions in white matter between male and female? Because many studies have found gender differences in brain white matter, I am curious whether perfusion in white matter can reflect certain properties?

Following this question, we investigated white matter perfusion using calibrated, partial volume–corrected data. We observed sex differences in white matter perfusion: females exhibited greater CBF than males across the lifespan. These sex differences emerged during development and persisted through aging. As expected, the average perfusion in white matter was lower than in cortical tissue. This figure is now added to the manuscript as *supplementary figure 1.*

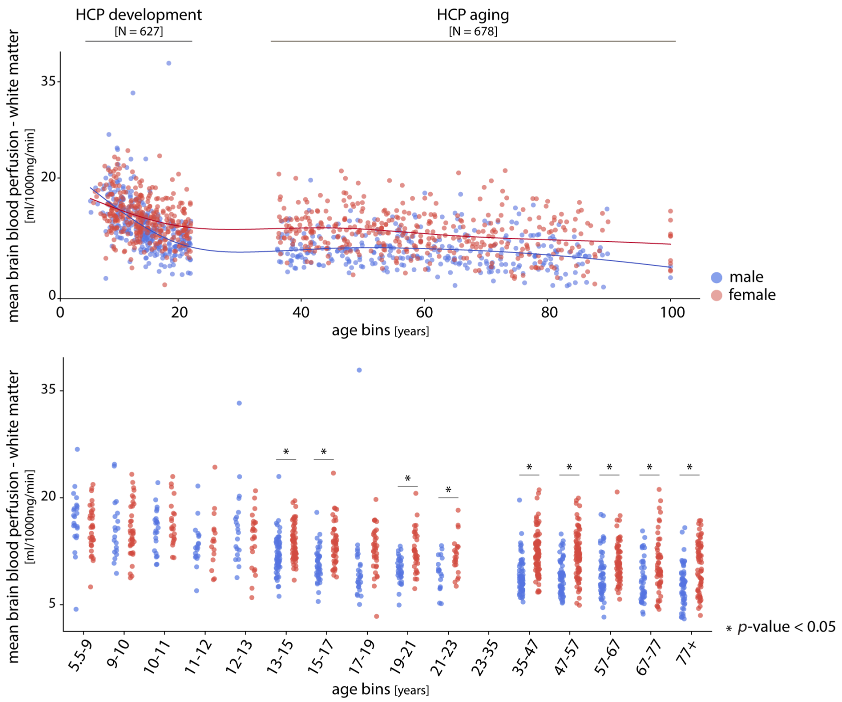

“**Figure S1. Mapping white matter blood perfusion across participants |** Each dot corresponds to a participant’s mean blood perfusion level within the white matter mask defined as part of the HCP ASL preprocessing pipeline (blue: male, red: female). Top: Sex-stratified generalized additive models for location, scale and shape (GAMLSS) [22] are used to model age-related changes in blood perfusion across the human lifespan. Bottom: Statistical comparisons of white matter blood perfusion between males and females are conducted within each age bin (13–15 years: t = −3.76, p = 2.62 × 10−4; 15–17 years: t = −4.97, p = 3.43 × 10−6; 19–21 years: t = −4.73, p = 1.36 × 10−5; 21–23 years: t = −2.96, p = 5.29 × 10−3; 35–47 years: t = −7.26, p = 1.75 × 10−11; 47–57 years: t = −6.69, p = 9.47 × 10−11; 57–67 years: t = −3.86, p = 1.88 × 10−4; 67–77 years: t = −3.39, p = 9.98 × 10−4; > 75 years: t = −4.61, p = 1.01 × 10−5; other age bins: p > 0.05).”

2) There is no description about whether the correlation between perfusion and gene expression is based on ROI or vertex? We know that the size of each ROI is different. AHBA map should be based on ROI. If you use the perfusion score map calculated based on vertex to do the correlation, will it be biased?

In the current version of the manuscript, the correlation analysis is performed between parcel-wise gene expression and parcel-wise perfusion scores. The gene maps are extracted and parcellated using the *abagen* toolbox. This is now clarified in the "*Methods/Cortical layer IV"* section of the manuscript:

> "Cortical layer IV pattern is estimated as the average expression map of 5 genes preferentially expressed in human granular layer IV. Gene maps for "COL6A1", "CUX2", "TRMT9B", "GRIK4"and "RORB" are obtained from *abagen* toolbox, publicly available at https://github.com/rmarkello/abagen/. Throughout the manuscript, we analyze gene maps parcellated using the Schaefer-400 atlas. Following Burt et al., each gene map is standardized using z-scoring, and their average is calculated to obtain the layer-specific cortical pattern."

We used the Schaefer-400 parcellation, which provides a more uniform parcel size distribution compared to alternatives like the Desikan-Killiany atlas. Given the sparsity of the Allen Human Brain Atlas (AHBA) sampling data, using a uniform parcellation helps minimize potential biases related to region size.

To clarify that the comparisons were done in the Schaefer parcellation, the Fig. 2c,d legend clarifies that the scatter-plot points denote Schaefer400 parcels.

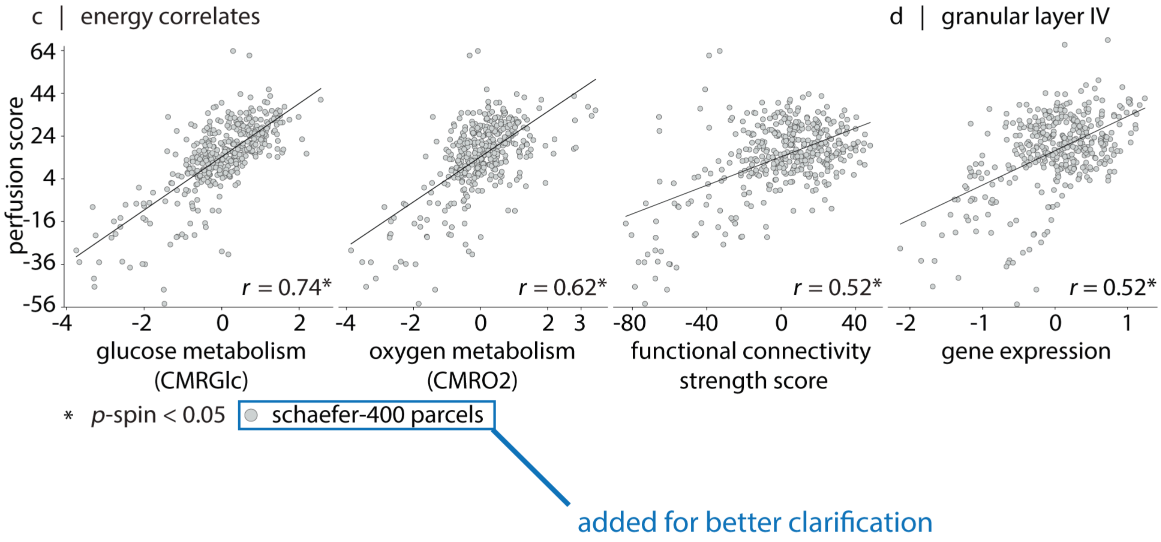

Finally, we note that the analysis could theoretically also be performed at the vertex level (although this was not done in the present manuscript). As proof of concept, we used the interpolated dense AHBA gene expression maps recently made available by Konrad Wagstyl et al. (2024). We extracted the vertex-level gene maps for layer IV, averaged them and correlated them with our voxel-wise perfusion map (Figure 2). The results of the analysis are shown below; we indeed observed an association between perfusion and gene expression patterns of layer IV.

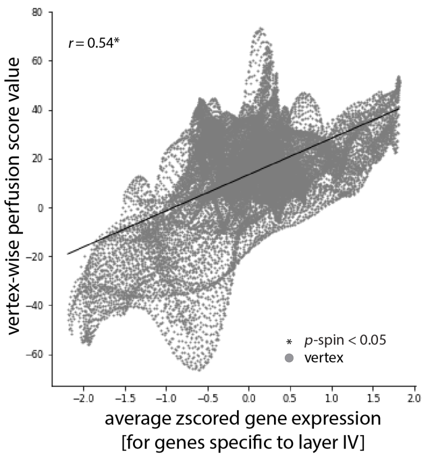

Reference:

Wagstyl, K., Adler, S., Seidlitz, J., Vandekar, S., Mallard, T. T., Dear, R., ... & Raznahan, A. (2024). Transcriptional cartography integrates multiscale biology of the human cortex. Elife, 12, RP86933.

Reviewer #2,
Peter Zhukovsky (note, reviewer 2 has signed this review):

The authors leverage HCP aging and development data (n>1000) across lifespan to provide a comprehensive analysis of cerebral blood perfusion. They leverage brain map-to-brain map associations to show that higher levels of perfusion are seen in regions with high metabolic demand and resting-state functional hubs. Using imaging transcriptomics with AHBA, they show that higher perfusion is seen in the granular layer IV, and correlative evidence suggests its related to GO pathways such as vasculogenesis and potassium and sodium transport and glucose. Interesting age and sex effects and associations with blood biomarkers are found. Sex effects appear to emerge around adolescence; common plasma proteins explain a lot of variance in the perfusion map in males and females separately. Overall, a lot of work and analyses have been completed for this project, with comprehensive visualizations and supplementary information. The link to neurodegenerative disorders would have been stronger if a dementia dataset with CBF/perfusion was included, ie analyzing individual CB perfusion maps from patients with varying levels of cognitive impairment, but it does not detract from the interesting insights into the anatomical patterns of perfusion distribution across the brain. The PLS analysis is one of the strongest aspects of this paper in my view. The implications of the tight coupling between common plasma biosignatures and CBF perfusion should be discussed more. I have several specific points below, mostly related to figures and minor issues, with one question on statistical reporting for Figure 4. While the manuscript is well written and structured overall, the writing style could be tightened up at times, e.g. rhetorical questions in the results or using 'Zooming in' in the discussion could be compressed for a more succinct writing style.

Dementia dataset:

With regard to the *Results* presented in the subsection called *Cerebral blood perfusion and susceptibility to neurodegeneration,* our aim is to discuss the hypothesis that regions with low intrinsic cerebral blood perfusion may be more susceptible to specific neurodegenerative conditions. Of course a more direct assessment of this hypothesis requires future studies involving patient-level perfusion data, we emphasize the importance of this phenomenon in the *Discussion*, stating:

> "Although these associations are derived from normative perfusion, they point to a pervasive influence of perfusion on healthy and non-healthy aging that should be studied more directly using perfusion measurements in individual patients."

To our knowledge, the Human Connectome Project (HCP) is the only open-access dataset that provides high-resolution (2.5 mm) Arterial Spin Labeling (ASL) data for over a thousand participants, using a pseudo-continuous imaging protocol (which is superior to single-pulse methods for CBF measurements). Given its unique advantages, we chose to focus exclusively on this dataset. Notably, the HCP is currently expanding to include data on neurodegenerative conditions. As soon as these data become available, we plan to conduct follow-up analyses to directly test our hypothesis. We also hope that our findings will motivate or support ongoing experimental work in this area.

Discussion on coupling between plasma biosignatures and CBF:

To further emphasize the coupling between plasma biomarkers and blood perfusion we have now added more explanation in the *Discussion* section (seventh paragraph), as below:

“Incorporating the HCP-Aging dataset, we also examine changes in cerebral blood perfusion during aging (36--100 years). Atherosclerosis, impaired cardiac output, and elimination of brain vasculature all affect cerebral blood perfusion and contribute to functional impairments in aging [citations]. We find greater age-related reduction in perfusion in border areas between major arterial territories, in line with the fact that these areas have reduced vascular density and smaller vessels and are vulnerable to embolism [citations]. Going further, we also show that additional physiological factors are associated with age-related reductions in perfusion, including mean arterial pressure (MAP), BMI, glucose, and HbA1c, reminiscent of the fact that metabolic syndrome exacerbates blood perfusion reduction, above and beyond the effect of sex and age [citations]. Indeed, high glucose (as well as obesity and insulin resistance) can lead to microangiopathy, atherosclerosis [1-2] and endothelial dysfunction through activation of protein kinase C, overexpression of growth factors and/or cytokines, and oxidative stress [3]. Meanwhile, our results point to the possible atheroprotective effect of HDL; HDL is involved in suppressing vascular-LDL accumulation, inflammation, and endothelial damage [4,5]. The tight coupling between plasma biomarkers and cerebral blood perfusion underpins the importance of considering body-brain interactions in the aging population.”

We have also added the following five references to the manuscript to support the added statements:

“1: McMillan, D. E. (1983). The effect of diabetes on blood flow properties. Diabetes, 32(Supplement_2):56–63.

2: Steinberg, H. O., Chaker, H., Leaming, R., Johnson, A., Brechtel, G., Baron, A. D., et al. (1996). Obesity/insulin resistance is associated with endothelial dysfunction. implications for the syndrome of insulin resistance. The Journal of clinical investigation, 97(11):2601–2610.

3: Calles-Escandon, J. and Cipolla, M. (2001). Diabetes and endothelial dysfunction: a clinical perspective. Endocrine reviews, 22(1):36–52.

4: Badimon, L. and Vilahur, G. (2012). Ldl-cholesterol versus hdl-cholesterol in the atherosclerotic plaque: inflammatory resolution versus thrombotic chaos. Annals of the New York Academy of Sciences, 1254(1):18–32.

5: Ouimet, M., Barrett, T. J., and Fisher, E. A. (2019). Hdl and reverse cholesterol transport: Basic mechanisms and their roles in vascular health and disease. Circulation research, 124(10):1505–1518.“

Writing style:

With regard to the writing style, we have revised the manuscript to improve the narrative; namely, we removed rhetorical questions from the *Results* section (see response to comment 3); and removed the phrase “Zooming in” from the *Discussion* section:

Before:

“Zooming in, we also find that spatial variation in perfusion mirrors spatial variation in cellular organization.”

Current version:

> "At a finer spatial resolution, we also find that spatial variation in perfusion mirrors spatial variation in cellular organization."

1 - Fig 4b the 2d map may not be necessary here given that its hard to make out which region is which on the 2d map - just keeping a larger version of the 3d brain maps would be better instead

Given that the data and analyses are all at the surface level, the flat maps provide an alternative visualization that will be more natural for some readers and potentially broaden the appeal of the manuscript.

Importantly, flat representations better preserve surface continuity compared to mid-thickness or inflated surfaces, which obscure regions buried within cortical folds and can lead to a loss of information about the buried surface area. The primary advantage of these 2D visualizations is that they allow the entire cortical surface to be viewed at once, with modest areal distortions and no perspective distortions associated with 3D surfaces viewed obliquely (Van Essen, 2005).

Reference:

> *Van Essen, D. C. (2005). A population-average, landmark-and surface-based (PALS) atlas of human cerebral cortex. Neuroimage, 28(3), 635-662.*

2 - Blood perfusion across development section - the authors need to report main effects vs interactions for age, sex and age x sex. The way the section is described right now suggests an age x sex interaction, whereby up to age 15 or so there are no sex differences, which then emerge and get stronger with age. That makes sense; however this conclusion should be supported by appropriate stats (ie interaction testing).

The table below (Table S4. in the manuscript) shows the statistical comparisons of blood perfusion between male and female participants across different age bins. The results indicate that sex differences begin to emerge around the 13–15 age bin and continue to exist in subsequent age groups.

Age Bin	#Male	#Female	t-statistic	p-value
<9	23	34	-0.8088	4.2529e-01
9-10	20	39	-0.5507	5.8469e-01
10-11	24	22	-0.9784	3.3330e-01
11-12	19	16	-0.4404	6.6357e-01
12-13	20	24	0.5778	5.6653e-01
13-15	64	62	-2.3889	1.8474e-02
15-17	42	46	-4.7678	7.5291e-06
17-19	27	36	-4.3597	5.2314e-05
19-21	33	36	-4.3922	4.1910e-05
21-23	18	22	-4.0244	2.6888e-04
29-35	0	0	–	–
35-47	67	98	-6.7027	3.9130e-10
47-57	65	88	-6.7457	3.0873e-10
57-67	58	66	-5.4027	3.5573e-07
67-77	47	61	-3.6007	4.8620e-04
>77	60	68	-3.9912	1.1106e-04

TABLE S4. **Comparison of male and female cerebral blood perfusion values in different age bins** | The t-statistics and p-values per each age-bin are shown. "#" denotes the number of participants.

Following the Reviewer's suggestion, we test the interaction between age and sex on cerebral perfusion during development. We fit a linear model including main effects of age, sex, and their interaction. The model was specified as:

Perfusion~Constant term + Age + Sex + Age×Sex (0: females and 1:males)

The results show:

- A significant main effect of age (β = -1.8116, p =2.3767e-27), indicating that perfusion changes across age.

- A significant sex difference at the intercept (β = 8.4246, p = 2.6424e-02), with males starting at a different baseline level.

- A significant interaction between age and sex (β = -1.0515, p = 3.2609e-05, suggesting that the age-related trajectory differs between sexes (steeper decline for male perfusion).

We have added the results of this analysis to the revised manuscript in the form of a supplementary figure (Fig. S12):

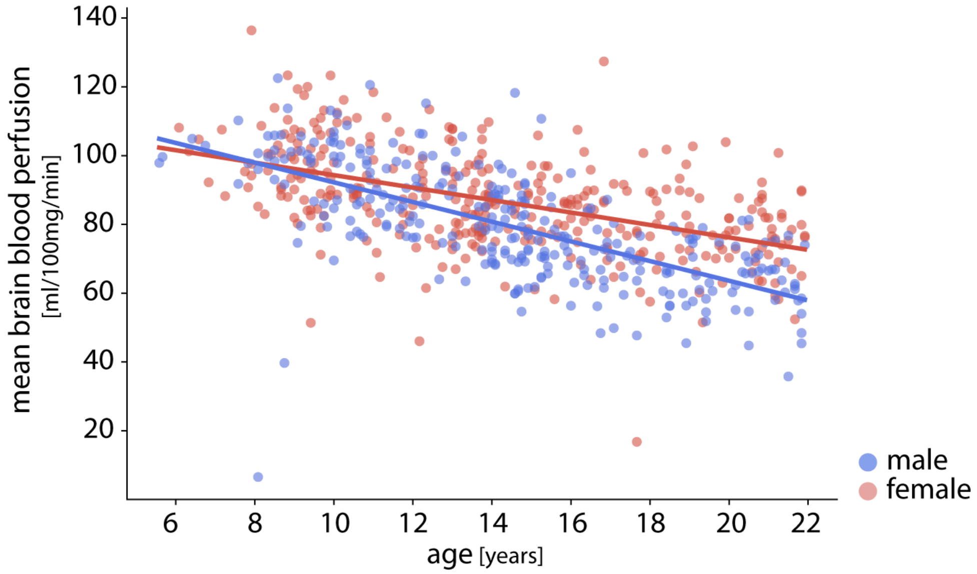

"**Figure S12. Sex differences in developmental perfusion trajectories |** To test the interaction between age and sex on cerebral perfusion during development, we fit a linear model including main effects of age, sex, and their interaction: perfusion = $\beta0 + \beta1 \times$ age + $\beta2 \times$ sex + $\beta3 \times$ age $\times$ sex (female = 0, male = 1; $\beta0$ = 112.44). The model reveals age-related decline in perfusion ($\beta1 = -1.81$, $p = 2.4 \times 10{-}27$). There is also a sex difference in baseline perfusion ($\beta2 = 8.42$, $p = 0.026$), and an interaction between age and sex ($\beta3 = -1.05$, $p = 3.26 \times 10{-}5$), suggesting that males exhibit a steeper age-related decline in grayordinates' perfusion compared to females (red: female; blue: male)."

- "Which brain regions experience the greatest developmental changes in blood perfusion?" - results should not include rhetorical questions

In *Results/Areal organization of blood perfusion* section, we change the question into a statement as:

> "We explore how perfusion co-localizes with canonical features of brain structure and function."

Also in Results/Blood perfusion across development section, the second paragraph, we change the question into a statement, as indicated below:

> "We then explore which brain regions experience the greatest developmental changes in blood perfusion."

4 - Figure 5 - there is a lot of white space; the brain image scaling and p-spin/atlas references could be improved slightly

We changed the legend of panel b from "cortical border-zones based on stroke-based atlas" into "*border-zones based on stroke-based atlas*"; and in panel c, we changed the legend from "parcels located in border-zone" into "*border-zones*".

We also repositioned the legends for panel c (moving it from the right side to below the panel).

The applied modifications reduced the white space. The updated version of figure 5 is included below:

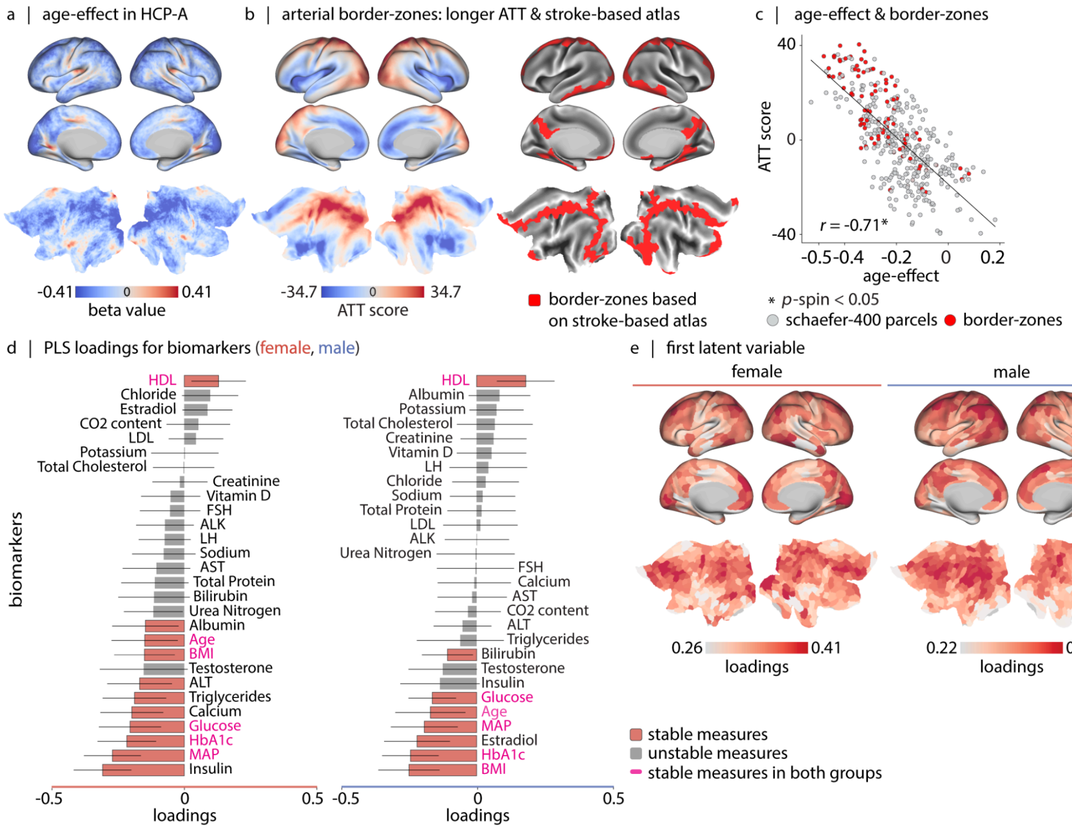

5 - Figure 5d - please don't call plasma proteins 'physiological measure', it's too general and is more commonly used to refer to heart rate and breathing monitoring during an fmri scan. Either plasma proteins or plasma biomarkers are much more appropriate here.

The PLS model also includes measures such as body mass index, age and blood pressure, which are not plasma-derived. We now have changed all instances of "physiological measures" to "biomarkers" throughout the manuscript. To clarify this point and improve specificity, we added more text to the *Methods/Dataset* section of the manuscript, as follows:

> "In addition to imaging data, the HCP-A dataset includes a comprehensive array of participant-specific blood laboratory results (plasma measurements) and bodily vitals. The plasma panel includes common health indicators such as total protein, glucose, insulin, hemoglobin A1c (HbA1c), triglycerides, low-density lipoprotein (LDL), high-density lipoprotein (HDL), total cholesterol, albumin, bilirubin, creatinine, urea, chloride, sodium, potassium, calcium, vitamin D, and CO2 content, as well as liver metabolic enzymes including alanine aminotransferase (ALT), aspartate aminotransferase (AST), and alkaline phosphatase (ALP). Multiple hormonal measures are also obtained including serum estradiol, testosterone, Luteinizing hormone (LH), and follicle-stimulating hormone (FSH). This extensive biochemical data is available for 597 out of 678 participants in the HCP-A dataset who have corresponding ASL data. Furthermore, participants' height and weight data are provided, enabling the calculation of body mass index (BMI). Systolic and diastolic blood pressure/pulse values are also recorded, from which the mean arterial pressure (MAP) is computed. Throughout the manuscript, we refer to the combination of plasma measurements, vitals (including BMI and MAP) and age as biomarkers. Fig. S19 presents scatter-plots illustrating the relationship between participants' raw measures and age."

In addition to the manuscript text, we have updated the language throughout the figures, for instance, in Figure 5,d now we have:

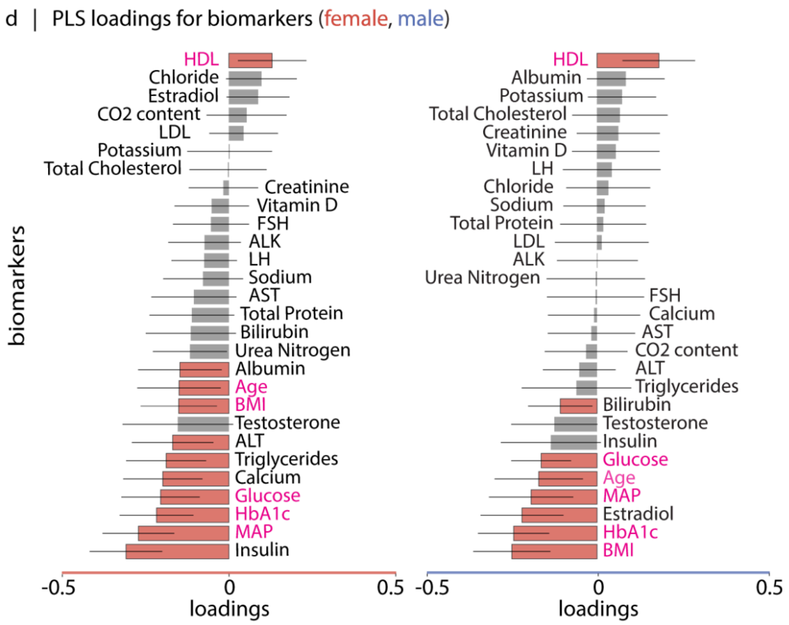

6 - Figure 5d - female estradiol is a key marker for the 'male' group? Seems contradictory, could the authors please comment?

Traditionally, testosterone and estrogen have been considered to be male and female sex hormones, respectively. However, estradiol, the predominant form of estrogen, is present in both sexes, with males having 10-50 pg/mL and premenopausal females having 10 to 300 pg/mL and postmenopausal women having 20-50 pg/mL in plasma. In men, estrogen is an important hormone for hypothalamic–pituitary–testicular axis regulation, growth hormone insulin-like growth factor-1 axis regulation, body composition and glucose metabolism [1]. To avoid the confusion that Estradiol is sex-specific (female-specific) we removed the term "*Female*" from the manuscript text and figures.

To answer this question and to better illustrate the data used in the PLS model, we have added a supplementary figure showing the raw PLS input data coming from the HCP-Aging cohort (the Estradiol panel is the third from the left, on the first row). We refer to this new supplementary information in the text. We have now added to following sentence to *Results/Blood perfusion across healthy aging* section:

> "These measures include blood laboratory results, age, mean arterial pressure (MAP), and body mass index (BMI). Blood laboratory parameters encompass total protein, glucose, insulin, hemoglobin A1c (HbA1c), triglycerides, low-density lipoprotein (LDL), high-density lipoprotein (HDL), total cholesterol, albumin, bilirubin, creatinine, urea, chloride, sodium, potassium, calcium, vitamin D, and CO2 content, liver metabolic enzymes (ALT, AST, ALP), and hormonal measures (estradiol, testosterone, LH, and FSH) (Fig. S20 shows the raw measurement values versus participants' age)."

We have also added a sentence to reference the new supplementary figure in the *Methods/Biomarkers* section; at the end of the first paragraph:

> "Fig. S20 presents scatter-plots illustrating the relationship between participants' raw measures and age."

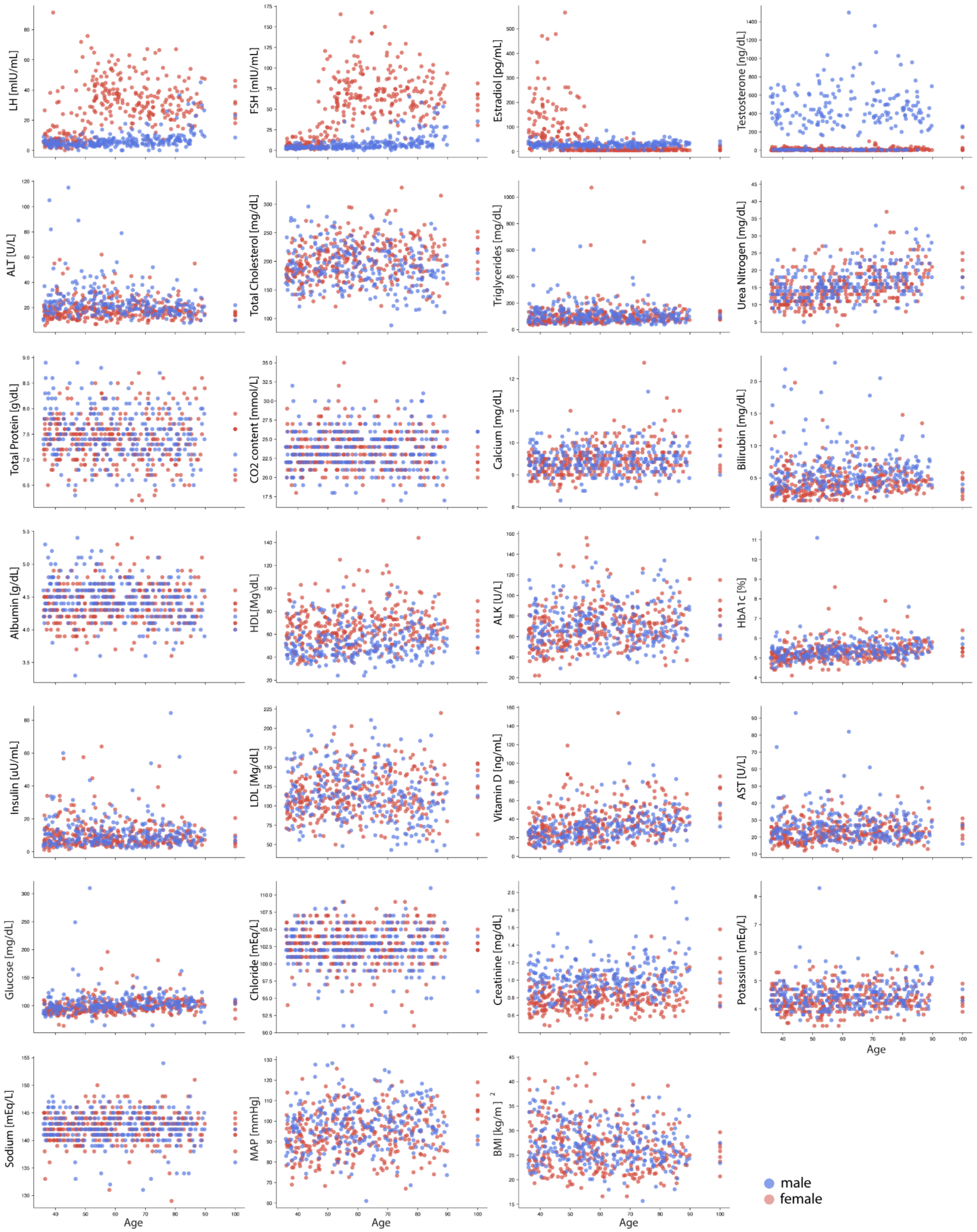

LH [mIU/mL]
FSH [mIU/mL]
Estradiol [pg/mL]
Testosterone [ng/dL]
ALT [U/L]
Total Cholesterol [mg/dL]
Triglycerides [mg/dL]
Urea Nitrogen [mg/dL]
Total Protein [g/dL]
CO2 content [mmol/L]
Calcium [mg/dL]
Bilirubin [mg/dL]
Albumin [g/dL]
HDL[Mg/dL]
ALK [U/L]
HbA1c [%]
Insulin [uU/mL]
LDL [Mg/dL]
Vitamin D [ng/mL]
AST [U/L]
Glucose [mg/dL]
Chloride [mEq/L]
Creatinine [mg/dL]
Potassium [mEq/L]
Sodium [mEq/L]
MAP [mmHg]
BMI [kg/m]2
Age
Age
Age
Age
male
female

The added figure caption reads as:

> "Fig. S20. **Biomarkers versus age |** Relationship between the raw biomarkers used in the PLS analysis (*y*-axis) and participants' age (*x*-axis) (red: female; blue: male)."

Specifically, if we zoom in on the changes in Estradiol values in male, we can see an increase in this measure in aging (rho = 0.15; p-permutation = 0.012; n-permutation = 1,000).

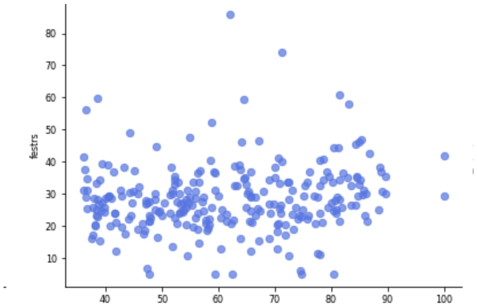

We observed that elevated estrogen levels may be related to reduced brain blood perfusion in men. However, to our understanding of literature, the role of estrogen in atherosclerosis remains inconclusive. Meanwhile, longitudinal studies in men suggest a possible relevance of elevation in Estradiol and Carotid intima-media thickness progression (a marker of early atherosclerosis) [2, 3]. As such, we decided to refrain from drawing definitive interpretations regarding this component while providing the information transparently to initiate further investigation on the clinically actionable information of estrogen in men's health.

References:

1: Russell, N., & Grossmann, M. (2019). Mechanisms in endocrinology: estradiol as a male hormone. European Journal of Endocrinology, 181(1), R23-R43.

2: Yeap, B. B. (2014). Sex steroids and cardiovascular disease. Asian journal of andrology, 16(2), 239-247.

3: Tivesten, A., Hulthe, J., Wallenfeldt, K., Wikstrand, J., Ohlsson, C., & Fagerberg, B. (2006). Circulating estradiol is an independent predictor of progression of carotid artery intima-media thickness in middle-aged men. The Journal of Clinical Endocrinology & Metabolism, 91(11), 4433-4437.

7 - Figure 7 and the associated analysis could be moved to supplementary if needed

We agree that this section can be a standalone project or a supplementary material; yet, we believe the results of this section (specifically the semantic mapping of vascular territories) might be informative for clinical readers. Given that the manuscript is under the word limit, for now we have elected to keep it in the main manuscript.

8 - Figure S3c - strength is misspelled

Thank you!. The error is corrected. The updated figure is included here:

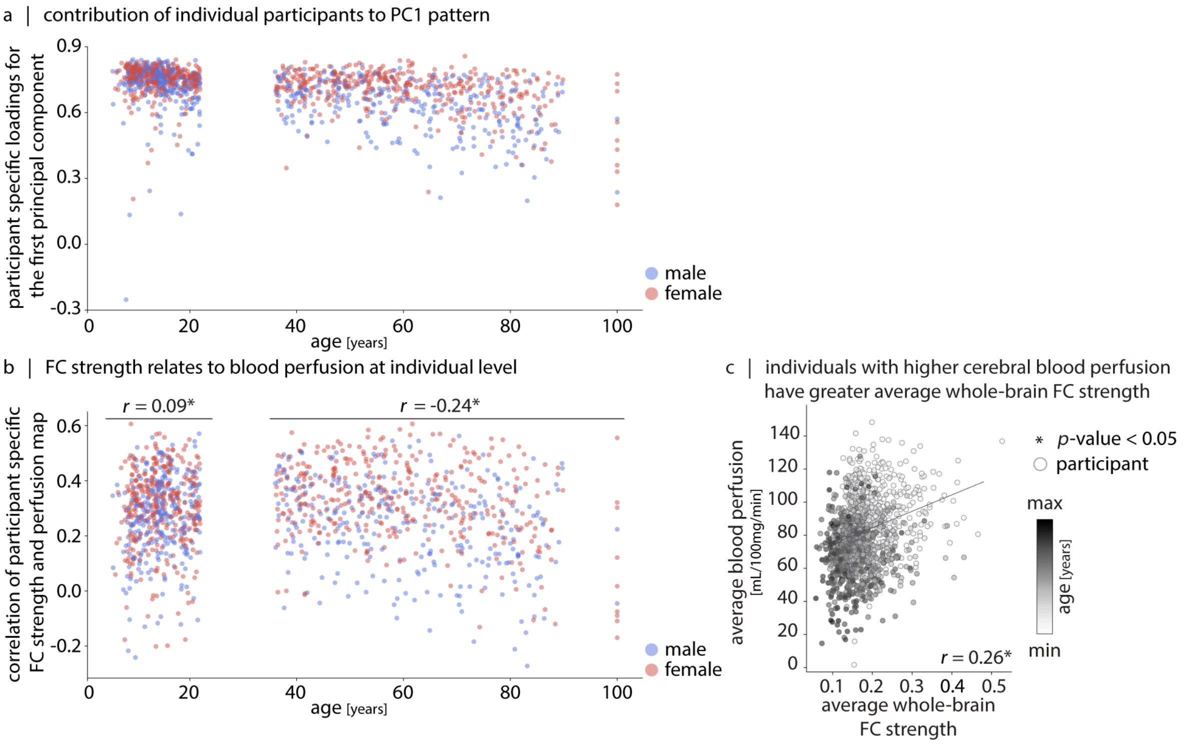

9 - Figure S4b - "comparison … <delete coming> from PET and ASL" for stylistic improvements

The word "*coming*" is removed from panel's b title. The updated figure is included here:

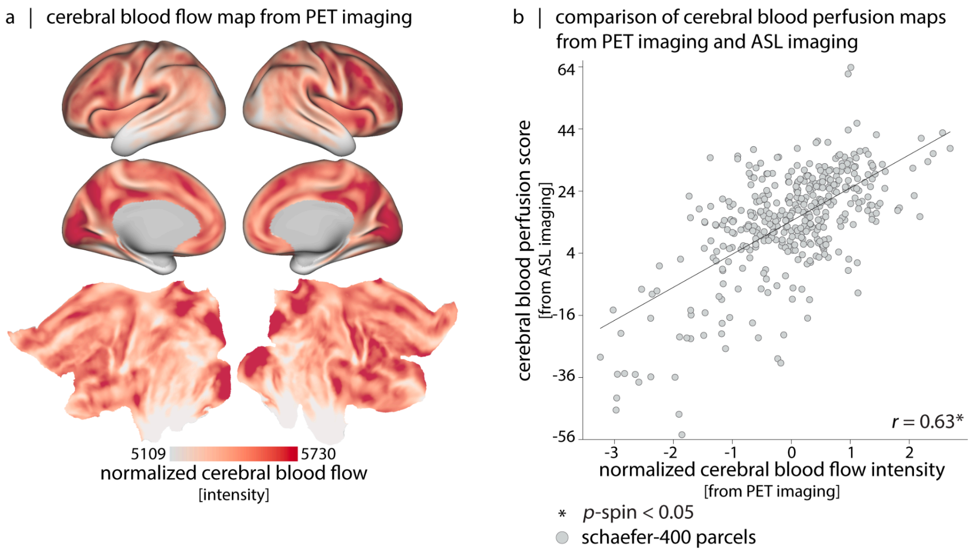

10 - Figure S13 - also please update physiological measures here

The change has been applied accordingly. The updated figure is included here:

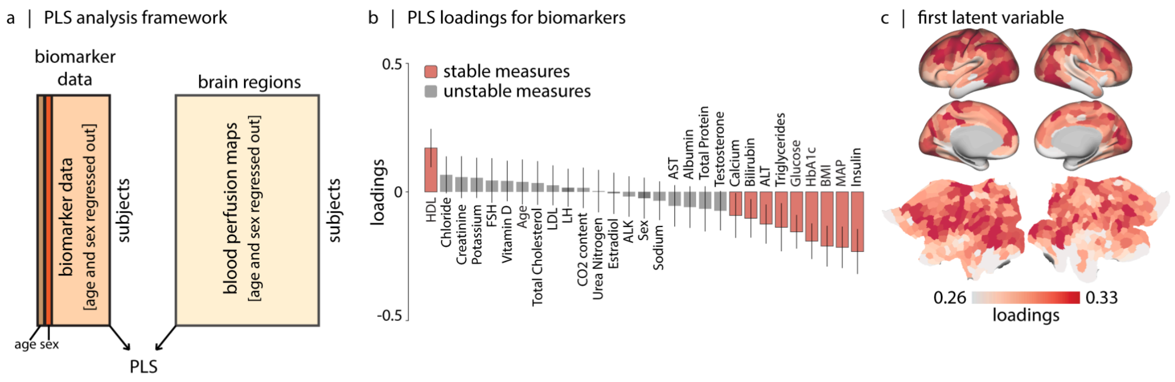

11 - For future PLS - if the authors are using pls bootstrap for robustness of weights, its worth reporting the weights as Z-scores to assess stability (see Morgan et al 2019 PNAS, Zhukovsky et al 2022 PNAS for code). Is it a PLS correlation or a PLS-regression? PLSC/PLSR makes this clear to the reader.

The results are coming from PLS-C (Krishnan et al., 2011). We now have stated this specifically in section *Methods/Partial least squares;* we have additionally stated the exact function we used to perform this analysis:

> "The partial least squares (PLS) component analysis is performed using the *behavioral_pls* function implemented in the *pyls* toolbox available at *https://github.com/netneurolab/pypyls/.* "

Reference:

> *Krishnan, A., Williams, L. J., McIntosh, A. R., & Abdi, H. (2011). Partial Least Squares (PLS) methods for neuroimaging: a tutorial and review. Neuroimage, 56(2), 455-475.*

Following the recommendation, here we show the *z*-score of weights following bootstrap resampling. As expected the results are similar to the reported findings in the manuscript.

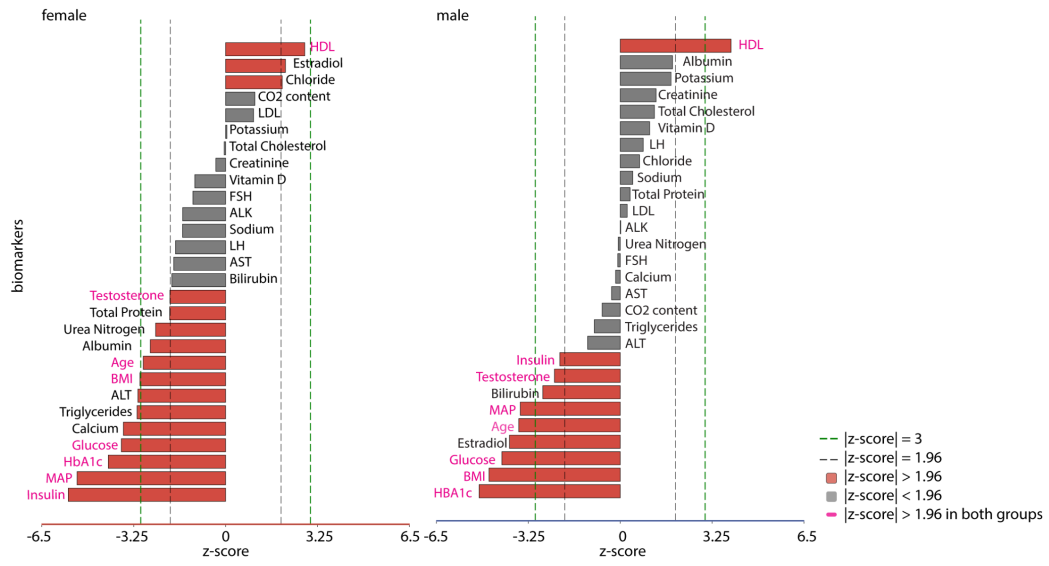

We added this information as the supplementary material to the manuscript, and added the stated references into the reference list of the manuscript as well. The figure caption reads as:

> "Fig. S21. **The z-scored weight distribution of biomarkers |** PLS bootstrapping is used to assess the robustness and stability of PLS weights [1,2]."

We now refer to this added supplement in Figure caption 5-panel d as:

> "(d)....The significance of each biomarker's contribution to the overall pattern is assessed by bootstrap resampling (1 000 bootstraps; see Fig. S21)."

The added references to the manuscript include the followings:

> *"1: Morgan, S. E., Seidlitz, J., Whitaker, K. J., Romero-Garcia, R., Clifton, N. E., Scarpazza, C., Van Amelsvoort, T., Marcelis, M., Van Os, J., Donohoe, G., et al. (2019). Cortical patterning of abnormal morphometric similarity in psychosis is associated with brain expression of schizophrenia-related genes. Proceedings of the National Academy of Sciences, 116(19):9604–9609*
>
> *2: Zhukovsky, P., Wainberg, M., Milic, M., Tripathy, S. J., Mulsant, B. H., Felsky, D., and Voineskos, A. N. (2022). Multiscale neural signatures of major depressive, anxiety, and stress-related disorders. Proceedings of the National Academy of Sciences,119(23):e2204433119."*

Reviewer #3:

This work analyzed pseudo-continuous arterial spin labeling (ASL) data from the HCP Lifespan studies (aged 5-22 years, and 36-100 years) to reconstruct a high-resolution normative cerebral blood perfusion map. The authors investigate how blood perfusion co-localizes with micro- and mesoscale features, including neurotransmitter signaling, cell types, and cortical layers. Then, the authors investigate how blood perfusion changes through the lifespan and in multiple neurodegenerative diseases. Finally, the authors show that interregional coordination of blood perfusion can be used to map arterial territories.
Overall, this work is very interesting and holds significant value for the field of neuroscience and research on brain development. However, I do have several major and minor concerns/issues, which are itemized below.

1. Cerebral blood perfusion is dynamic and changes throughout the lifespan. To quantify the effect of age on cerebral blood perfusion during development, the authors constructed linear regression models. However, the development process is not always linear (Bethlehem Richard AI, et al. Nature 2022). A generalized additive model for location, scale, and shape (GALSS), a robust method for modeling nonlinear growth trajectories, would be useful for constructing the development process across the human lifespan.

We have now added the use of GAMLSS models to describe the changes to the manuscript. Specifically, in Fig. 1, when we show the fitted trajectories across both the development and the aging cohort we use GAMLSS model:

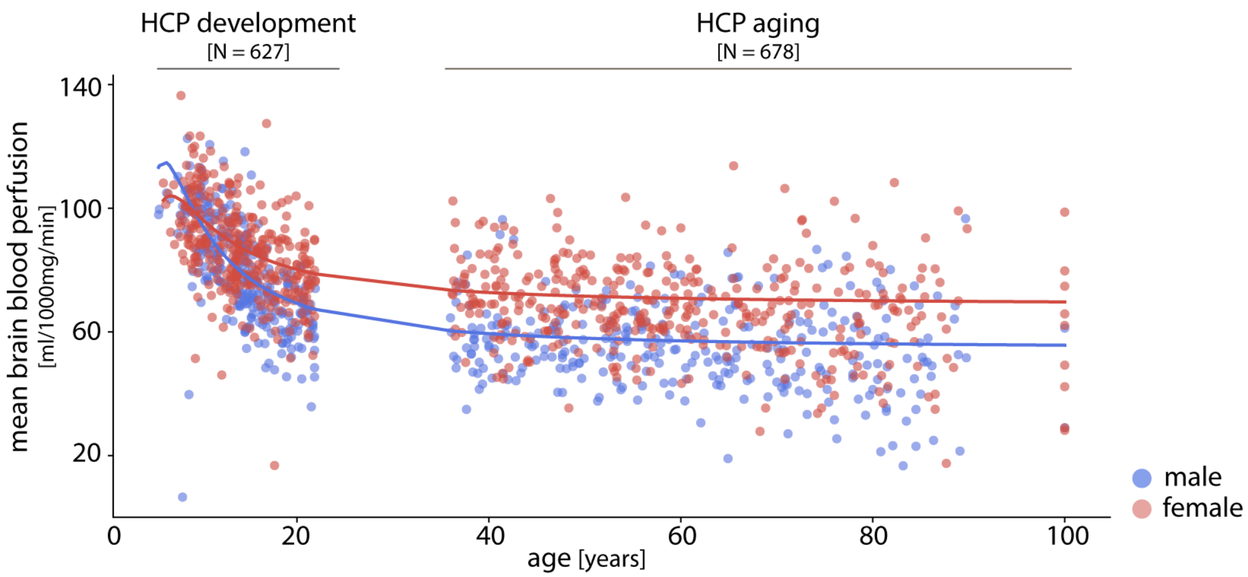

The figure caption for has also changed accordingly to reflect this update:

> "**Figure 1. Mapping cerebral blood perfusion across participants |** Cerebral blood perfusion is estimated using arterial spin labeling (ASL) from the HCP Lifespan studies, including 627 participants (337 females) from the HCP-D (development) and 678 participants (381 females) from the HCP-A (aging). Each dot corresponds to a participant's mean brain (grayordinates) blood perfusion level (blue: male, red: female)

(See Fig. S1 for the mean perfusion trajectory within white matter mask. Sex-stratified generalized additive models for location, scale and shape (GAMLSS) [1] are used to model age-related changes in blood perfusion.

We have also added a section to the *Methods* section named "*Generalized additive model for location, scale and shape*" to describe the model:

"**Generalized additive model for location, scale and shape**

Generalized additive model for location, scale and shape (GAMLSS) is implemented using the *gamlss* R package available at *https://github.com/gamlss-dev/* [2]. We use a sex-stratified GAMLSS modeling approach to characterize nonlinear normative cerebral blood perfusion trajectories in development and aging. Here cerebral perfusion in a given parcel or across the whole brain is assumed as a random variable (Y) following the generalized gamma (GG) distribution:

$$Y \sim \mathrm{GG}(\mu, \sigma, \nu) \qquad (1)$$

here, GG distribution parameters—location (μ), scale (σ), and shape (v)—can be modeled as functions of explanatory variable (age). In our analysis, μ and σ are modeled as the fractional polynomial functions of the explanatory variable, and v is set as a constant. Specifically, we used the `fp()` function to determine the best-fitting two-term fractional polynomials from the predefined set of power values: –2, –1, –0.5, 0, 0.5, 1, 2, 3. The optimal set of powers is chosen automatically through iterative model fitting to best capture nonlinear relationships between age and distribution parameters."

The added citations include:

"*1: Bethlehem, R. A., Seidlitz, J., White, S. R., Vogel, J. W., Anderson, K. M., Adamson, C., ... & Schaare, H. L. (2022). Brain charts for the human lifespan. Nature, 604(7906), 525-533.*

2: Stasinopoulos, D. M., & Rigby, R. A. (2008). Generalized additive models for location scale and shape (GAMLSS) in R. Journal of Statistical Software, 23, 1-46."

We also used GAMLSS models to characterize parcel-wise changes in cerebral perfusion across development and aging, in each of the Schaefer-400 parcels. The results are presented in response to *Point 2.*

2. The physical meaning of age coefficients (beta) of age-related changes is difficult to comprehend. To provide insight into the overall magnitude and direction of regional age effects, many previous studies (such as Sydnor Valerie, et al. Nature neuroscience, 2023; Li Jiao, et al. Plos Biology, 2024; Lianglong Sun, et al. Nature neuroscience, 2025) have represented the age effects (R2 values) as the age-related regional changes during development. Therefore, I suggest replacing the t-statistic with the age effect (R2 values).

To address this point and make the manuscript more comprehensive, we have added several new analyses to the manuscript. Specifically, we modeled developmental changes in cerebral blood perfusion

using a sex-stratified GAMLSS approach, as outlined in Bethlehem *et al.* In this regard, we have now added a figure "*Fig.S14*" to the paper, where:

> In *panel a,* each dot represents a subject's whole-brain mean perfusion, and we fit separate GAMLSS models for males and females.
> In *panel b,* we fit GAMLSS models to perfusion changes in development for each of the Schaefer-400 parcels; the obtained trajectories are shown here.
> In *panel c,* the R^2 values of the models are shown, with higher values indicating more structured age-effect.
> In *panel d,* we compare the average R^2 across Yeo networks, stratified by sex, showing that transmodal regions tend to have higher model fits compared to unimodal regions, with overall higher R^2 values observed in males.

This information is added to the revised manuscript, as described in the following paragraphs.

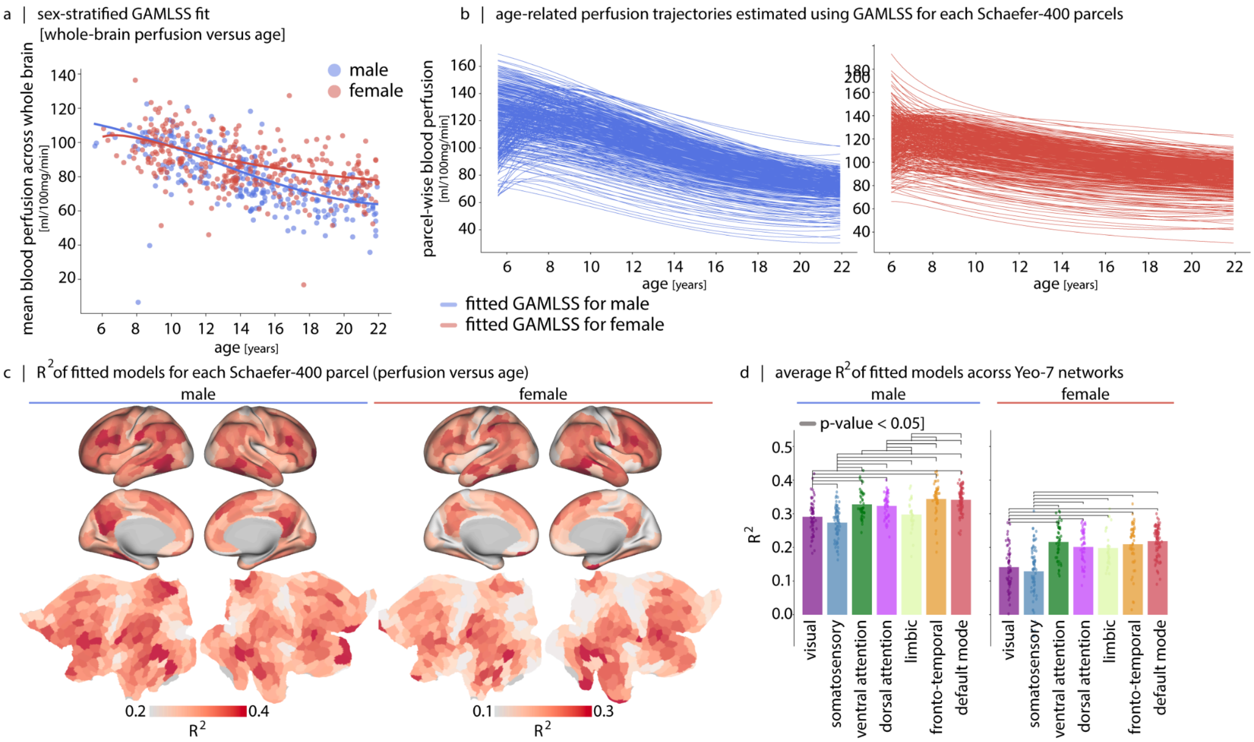

"**Figure S14. GAMLSS trajectories to model blood perfusion (versus age) in development |** (a) Each dot corresponds to a participant's mean brain blood perfusion level in the HCP-D cohort (blue: male, red: female). A separate GAMLSS model is fitted for each sex-group to capture age-related changes in grayordinates' perfusion during development. (b) Parcel-wise trajectories of cerebral blood perfusion across age are modeled using GAMLSS in each of the 400 Schaefer parcels. Each panel displays the fitted trajectories for male and female participants. (c) Model fit values are shown on lateral and medial views of the inflated and 2D cortical flat surfaces (fsLR). Higher values indicate better fits. (d) Comparison of mean model fit values across seven canonical intrinsic functional networks introduced by Yeo et al. shows that transmodal regions

exhibit higher model fits than unimodal regions. Additionally, model fits are higher in males than in females.“

We have also included Fig. S15, which displays the fitted GAMLSS trajectories of cerebral perfusion across all Schaefer-400 parcels, separately for each sex and stratified by Yeo network:

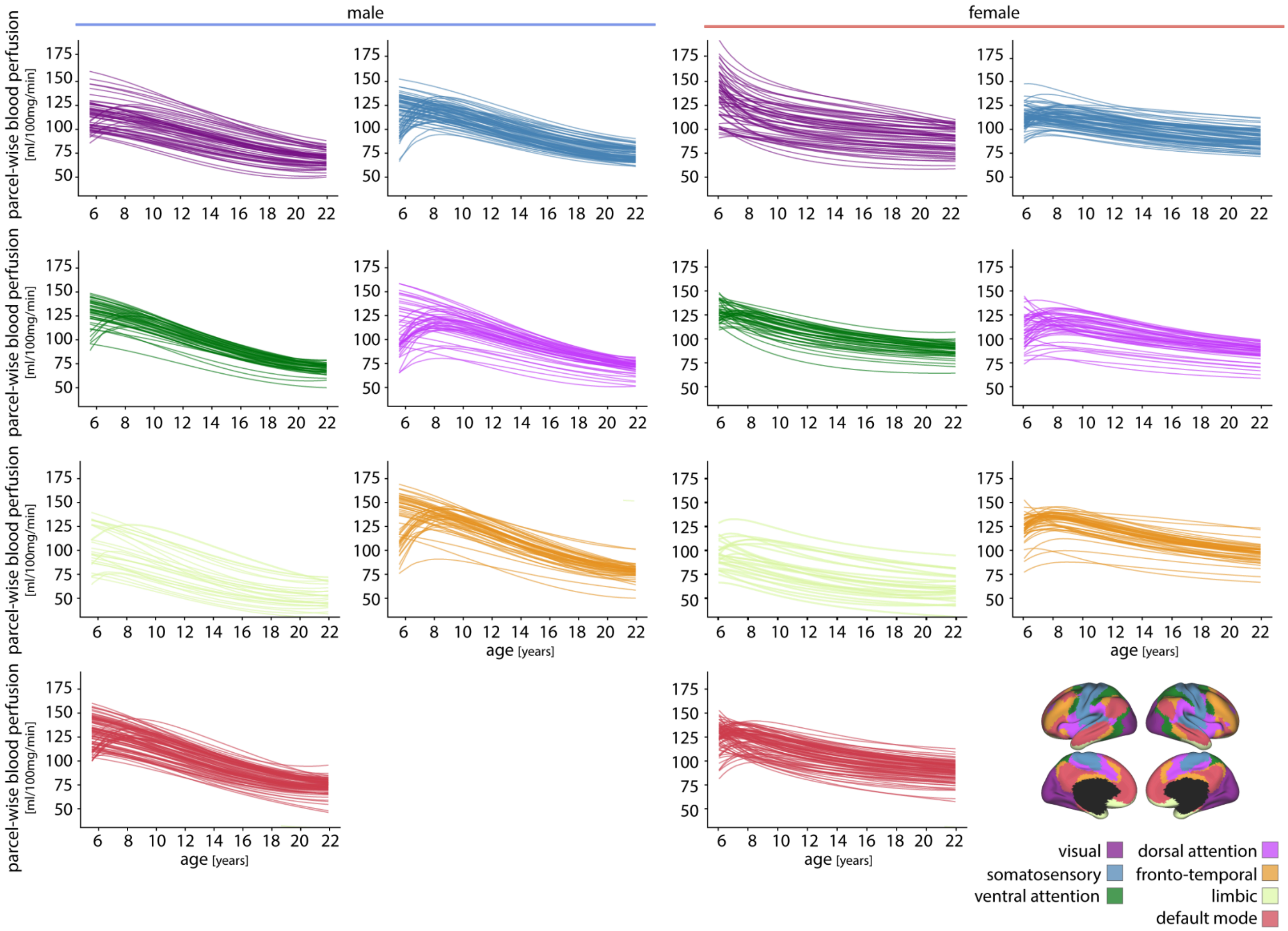

“**Figure S15. Sex-stratified trajectories of perfusion during development |** Fitted GAMLSS trajectories of cerebral perfusion are shown for each of the 400 Schaefer parcels, grouped by seven canonical intrinsic functional networks introduced by Yeo et al.“

In the manuscript text, specifically within the *Results/Blood perfusion across development* section, we refer to added analysis and results as follows:

“For completeness, we also used GAMLSS models to build sex-stratified trajectories of perfusion at both whole-brain (grayordinates) and parcel-level (Schaefer-400 parcels) scales (Fig. S14, Fig. S15). GAM models can capture non-linear age effects and are of interest in lifespan [1,2,3] and specifically developmental neuroimaging studies [4,5]. Using this approach, we replicate the greater age-related perfusion changes in transmodal regions during development (5-22 years).”

Also in *Figure caption of Fig. 4*, we added a sentence to refer to this results:

> “(b) Age-effect perfusion map estimated using data from 627 participants in the HCP-Development (5–22 years) dataset. Linear age coefficients (β1) are derived from a linear model (CBFi = β0 + β1 × age + β2 × sex), applied at each vertex/voxel i. Subcortical age-effect values are shown in Fig. S13a. Sex-effect and coefficient significance maps are provided in Fig. S10. See Fig. S14 and Fig.S15 for sex-stratified non-linear modeling of perfusion changes.“

As mentioned in our response to *point 1*, the explanation for the modeling is also added to the *Methods* section of the manuscript, under a section called “*Generalized additive model for location, scale and shape*”.

For completeness, we also use the same modeling approach for the aging cohort. The added supplementary information (Fig. S18) is included here:

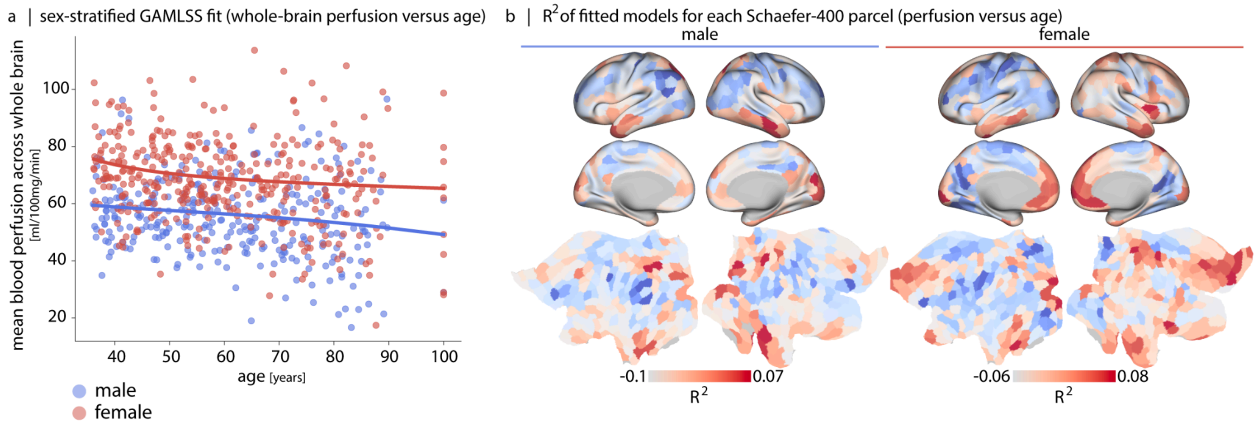

> “**Fig. S18. GAMLSS trajectories to model blood perfusion (versus age) in aging |** (a) Each dot corresponds to a participant's mean brain blood perfusion level in the HCP-A cohort (blue: male, red: female). A separate GAMLSS model is fitted for each sex-group to capture age-related changes in grayordinates’ perfusion during aging. (b) Parcel-wise trajectories of cerebral blood perfusion across age are modeled using GAMLSS in each of the 400 Schaefer parcels. Model fit values are shown on lateral and medial views of the inflated and 2D cortical flat surfaces (fsLR). Greater values indicate better fits.“

We also refer to this supplementary information in the manuscript text, more specifically within *Fig. 5. Caption*:

> “(a) Age-effect perfusion map estimated using data from 678 participants in the HCP-Aging dataset(36–100 years). Linear age coefficients (β1) are derived from a linear model (CBFi = β0 + β1 × age + β2 × sex), applied at each vertex/voxel i. Subcortical age-effect values are shown in Fig. S13b. Sex-effect and coefficient significance maps are provided in Fig. S16. Fig. S17 shows vertex/voxel-wise Spearman correlation maps of blood perfusion and age, stratified by biological sex. See Fig. S18 for sex-stratified non-linear modeling of perfusion changes.“

The References included for the added sections are listed below:

1: Bethlehem, R. A., Seidlitz, J., White, S. R., Vogel, J. W., Anderson, K. M., Adamson, C., ... & Schaare, H. L. (2022). Brain charts for the human lifespan. Nature, 604(7906), 525-533.

2: Sun, L., Zhao, T., Liang, X., Xia, M., Li, Q., Liao, X., ... & DIDA-MDD Working Group. (2024). Functional connectome through the human life span. Biorxiv, 2023-09.

3: Li, J., Zhang, C., Meng, Y., Yang, S., Xia, J., Chen, H., & Liao, W. (2024). Morphometric brain organization across the human lifespan reveals increased dispersion linked to cognitive performance. Plos Biology, 22(6), e3002647.

4: Stasinopoulos, D. M., & Rigby, R. A. (2008). Generalized additive models for location scale and shape (GAMLSS) in R. Journal of Statistical Software, 23, 1-46.

5: Sydnor, V. J., Larsen, B., Seidlitz, J., Adebimpe, A., Alexander-Bloch, A. F., Bassett, D. S., ... & Satterthwaite, T. D. (2023). Intrinsic activity development unfolds along a sensorimotor–association cortical axis in youth. Nature Neuroscience, 26(4), 638-649.

3. The Results section contains too much description of the Methods. The authors should mainly describe the results and what they reveal.

We thank the reviewer for the comment, and as indicated in response to reviewer 2, point 3, we have modified some statements in the *Results* section to streamline the descriptions of the analyses. However, in alignment with the editor's guidance, we have retained some description of analytical procedures within the *Results* section; as the journal encourages comprehensive reporting in this part of the manuscript. We hope the reviewer finds this approach acceptable.

4. The authors used ALS data from the HCP-D and HCP-A datasets to investigate age-related changes in cerebral blood perfusion. However, data from individuals aged 23 to 35 years are missing. Therefore, the use of the term "lifespan" in the title is misleading and not appropriate. The term "HCP Lifespan studies (5-100 years)" should be revised to reflect the actual age coverage of the datasets used.

We changed the manuscript title from "***Cerebral blood perfusion across biological systems and the human lifespan***" into "**Cerebral blood perfusion across development and aging: Links with multi-scale organization of the human brain**".

To be more accurate, we have revised all instances of "***5-100 years***" in the manuscript to "***5–22 and 36–100 years***". This change has been applied consistently throughout the paper. Specifically, the abstract now read:

> "Here we analyze pseudo-continuous arterial spin labeling (ASL) data from 1 305 healthy individuals in the HCP Lifespan studies (5–22 and 36–100 years) to reconstruct a high-resolution normative cerebral blood perfusion map."

5. The authors estimate the cortical Layer IV pattern using the average expression map of five genes preferentially expressed in the human granular layer IV. However, the rationale for focusing exclusively on Layer IV-specific gene expression remains unclear. Prior work (e.g., Burt et al., 2018) has demonstrated layer-specific gene expression across three broader laminar groups: Layers 1-3, Layer 4, and Layers 5-6. To strengthen the biological interpretability of the findings, I recommend that the authors provide a justification for the selective focus on Layer IV, and, if feasible, consider incorporating gene expression patterns from additional cortical layers to offer a more comprehensive characterization of laminar contributions.

We originally focused on layer IV because it is the principal input layer of the cortex. This layer is known to be the most highly vascularized and is marked by the greatest density of mitochondrial cytochrome oxidase (Wong-Riley, 1989). The rationale for this choice is included below:

> "Specifically, we test whether perfusion is spatially correlated with granular layer IV, the primary input layer of the cortex. Fig. 2d shows the spatial correlation between ASL-estimated perfusion and the average expression map of five genes preferentially expressed in this layer [31, 274]. The observed positive correlation with perfusion (r = 0.52, pspin = 2.99×10−3) is consistent with previous imaging studies reporting denser regional vasculature of primary sensory areas and specifically cortical layer IV in human [63, 102, 227], macaque monkey [3, 13, 124], cat [275], rat [129] and mouse brain [26, 132, 267]; suggesting that the early stages of feed-forwarding processing are highly resource demanding. Collectively, these results build on animal and region-of-interest studies, showing that perfusion and areal differentiation are closely intertwined at the whole-brain level."

Nevertheless, following the Reviewer's suggestion, we have revised the manuscript to be more comprehensive by also examining the relationship between the blood perfusion map and transcriptomic signatures of layers I–III and V–VI. Following Burt et al., we used 13 gene markers of layers I–III ('C1QL2', 'C20orf103', 'CARTPT', 'DISC1', 'GLRA3', 'GSG1L', 'IGSF11', 'INPP4B', 'MFGE8', 'PVRL3', 'RASGRF2', 'SV2C',' WFS1'), and 28 gene markers of layers V–VI ('ADRA2A', 'AKR1C3', 'ANXA1', 'B3GALT2', 'CDH24', 'CTGF', 'ETV1', 'FAM3C', 'FOXP2', 'HTR2C', 'KCNK2', 'NPY2R', 'NR4A2', 'NTNG2', 'OPRK1', 'PCDH17', 'PCDH20', 'PCP4', 'PDE1A', 'RPRM', 'RXFP1', 'SNTB1', 'SYT10', 'SYT6', 'TLE4', 'TOX', 'TRIB2', 'VAT1L'). From these lists, three genes from the I–III group ('DISC1', 'PVRL3', 'C20orf103'), and four genes from the V–VI group ('NPY2R', 'TRIB2', 'CDH24', 'FAM3C') did not meet the differential stability threshold of 0.1 and were excluded from the analysis. We computed the mean expression map for each layer after removing these low-stability genes. Associations between the perfusion map and the transcriptomic signatures of layers I–III and V–VI were not statistically significant, unlike the significant association observed for layer IV. We thank the Reviewer for this suggestion, as it helps to show that the hypothesized associations with Layer IV, in addition to being statistically significant, are also specific. These additional results are now included in the supplementary information.

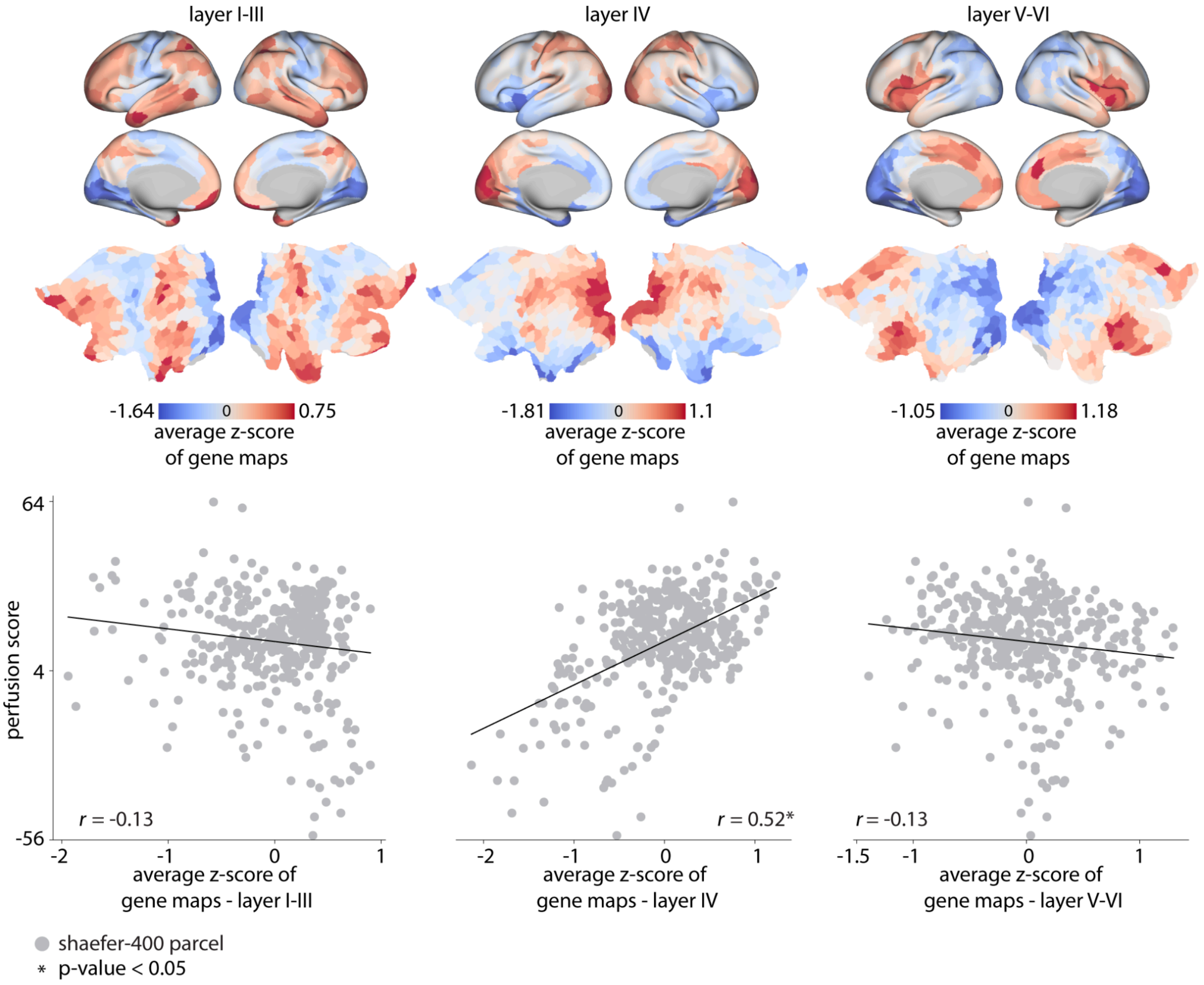

"Fig. S8. **Relating perfusion and laminar differentiation |** The top row shows the average z-scored transcriptomic signature of cortical layer I-III, IV, and V-VI, respectively [Burt et al., 2018]. The bottom row presents scatter plots with perfusion scores on the *x*-axis and the average z-score of gene maps for each layer on the *y*-axis. For layer I-III, the transcriptomic signature is defined using 13 genes (C1QL2, C20orf103, CARTPT, DISC1, GLRA3, GSG1L, IGSF11, INPP4B, MFGE8, PVRL3, RASGRF2, SV2C and WFS1). However, three of these genes (C20orf103, PVRL3 and DISC1) do not meet the differential stability of 0.1 and hence are excluded from the analysis. The transcriptomic signature for layer IV is based on 5 genes (COL6A1, CUX2, TRMT9B, GRIK4 and RORB), all of which pass the differential stability threshold of 0.1. For layer V-VI, there are 28 gene markers (ADRA2A, AKR1C3, ANXA1, B3GALT2, CDH24, CTGF, ETV1, FAM3C, FOXP2, HTR2C, KCNK2, NPY2R, NR4A2, NTNG2, OPRK1, PCDH17, PCDH20, PCP4, PDE1A, RPRM, RXFP1, SNTB1, SYT10, SYT6, TLE4, TOX, TRIB2 and VAT1L). Four of these genes (CDH24, FAM3C, NPY2R and TRIB2) do not meet the differential stability of 0.1 and are excluded from the analysis."

We also add the reference to this supplementary material in the manuscript text. Specifically, in *Figure 2 caption*, we have now cited the added figure:

> "...(d) The transcriptomic signature of cortical layer IV [Burt et al.] is positively correlated with perfusion (r=0.52, p-spin = 2.99 × 10−3). We do not find correspondence between blood perfusion and transcriptomic signature of infragranular (V–VI) and supragranular (I–III) cortical layers (Fig. S8)."

References:

Wong-Riley, M. T. (1989). Cytochrome oxidase: an endogenous metabolic marker for neuronal activity. Trends in neurosciences, 12(3), 94-101.

6. The authors utilize two cortical parcellation atlases—the Glasser and Schaefer-400 functional atlas. However, the rationale for using the Glasser atlas to quantify cortical perfusion scores, while employing the Schaefer-400 atlas for subsequent spatial association analyses remains unclear. This inconsistency may affect the interpretability and comparability of results across different parts of the study. I recommend that the authors clarify their reasoning for choosing different atlases and, if feasible, consider using the same atlas consistently throughout the analyses to enhance methodological coherence.

To clarify, all quantitative analyses presented in the manuscript—including the spatial correlations with micro- and meso-scale brain features—are conducted using the Schaefer-400 functional atlas. We selected this parcellation because of its more uniform parcel size distribution, which reduces parcel-size-related biases in spatial correlation analyses. To support this rationale, we have included a histogram that shows parcel size distributions of the Glasser and Schaefer atlases:

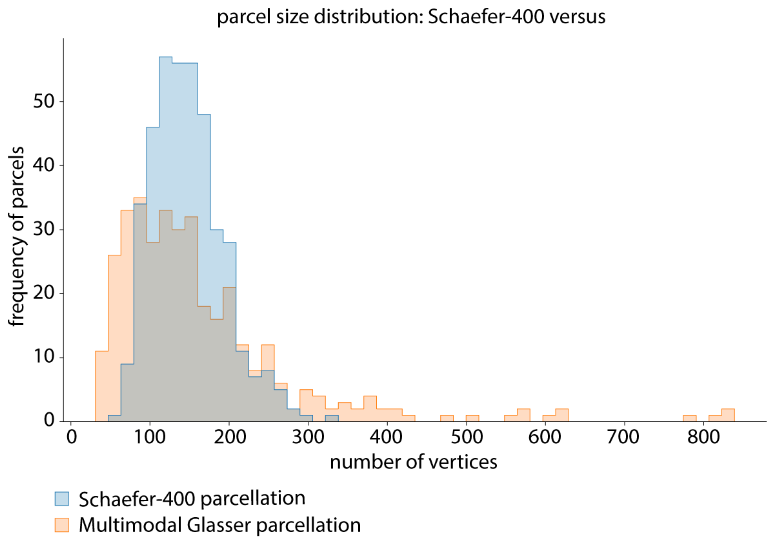

In our manuscript, the Glasser multimodal parcellation is used solely for area border visualization purposes in Figure 2a, and for reporting areal mean perfusion in Table S1. We chose the Glasser atlas for these specific purposes because its parcel labels correspond more closely to traditionally defined cortical areas, and its boundaries align more accurately with visuotopic and architectonic divisions of the cortex (Schaefer et al., 2018). We now state the rationale in the *Methods/Atlases* section of the manuscript:

“For all parcel-wise cortical analysis, we apply the Schaefer-400 functional atlas [1].

For quantifying cortical perfusion scores (Table.S1), we use the multi-modal Glasser parcellation; this parcellation divides each hemisphere into 180 distinct parcels based on function, cortical microstructure, and connectivity (Glasser et al., 2016). We used this parcellation only when showing areal borders in Fig.2a. This is because the Glasser atlas integrates multi-modal information and offers a more anatomically precise representation of cortical architectonic boundaries than the Schaefer atlas, which focuses solely on resting-state functional signal homogeneity [1]. Table.S1 and Fig.2a are the only instances where multi-modal Glasser parcellation is used.

For quantifying subcortical perfusion scores (Fig.S7), age-effect (Fig.S13), and perfusion covariance gradients (Fig.S23), we use the Tian functional atlas (S4 version; including 54 parcels; see Table.S2) (Tian et al., 2020).”

Reference:

Schaefer, A., Kong, R., Gordon, E. M., Laumann, T. O., Zuo, X. N., Holmes, A. J., ... & Yeo, B. T. (2018). Local-global parcellation of the human cerebral cortex from intrinsic functional connectivity MRI. Cerebral cortex, 28(9), 3095-3114.

7. The authors assess the associations between cortical perfusion scores and micro- and macros-scale features (such as oxygen metabolism, laminar differentiation, functional connectivity strength, gene expression, cell types, neurotransmitter receptors, and neuropeptide receptors). However, it is unclear whether subcortical structures (e.g., those defined by the Tian functional atlas) were excluded from these analyses.

We make the data for subcortical areas—such as the perfusion score maps, age effects, and vascular territories— available in the GitHub repository associated with this study and we also include these data in the supplementary information for potential use by other investigators. However, throughout the manuscript, all statistical analyses assessing the relationship between blood perfusion (or its age-related effects) and other brain features—including oxygen metabolism, laminar differentiation, functional connectivity strength, gene expression, cell types, neurotransmitter receptors, and neuropeptide receptors—are limited to cortical regions. This decision was made because the original sources for some of these features, such as oxygen and glucose metabolism, only provide cortical data in *fsaverage* surface format. Moreover, laminar differentiation is a property specific to cortical tissue. To avoid any ambiguity regarding the inclusion of subcortical structures, we consistently excluded them from statistical comparisons while still including them in descriptive summaries (Fig.S7, S13, S23) and publicly shared data. We also add a point to the limitations section of the *Discussion* (last paragraph*)* to emphasize the fact that we did not focus on subcortical structures in this paper:

“…Third, when estimating the association of perfusion with other biological features, age-related changes of perfusion in development and aging, and relating vascular territories to functional annotations, we focused on cortical regions. However, we provide quantification of subcortical perfusion values, age-related effects and perfusion covariance gradient values, and make the whole-brain maps openly available for further subcortex-focused analysis.“

8. It would be interesting to explore the relative contributions of these micro- and macro-scale features to cortical perfusion scores beyond simple spatial correlations—for example, through multivariate modeling and dominance analysis.

We fit two multi-linear regression models to predict blood perfusion patterns from (1) receptor distributions (neuropeptides and neurotransmitters) and cortical layer patterns (a,b). (2) only receptor distributions (neuropeptides and neurotransmitters) (c,d). The added supplementary information is included here:

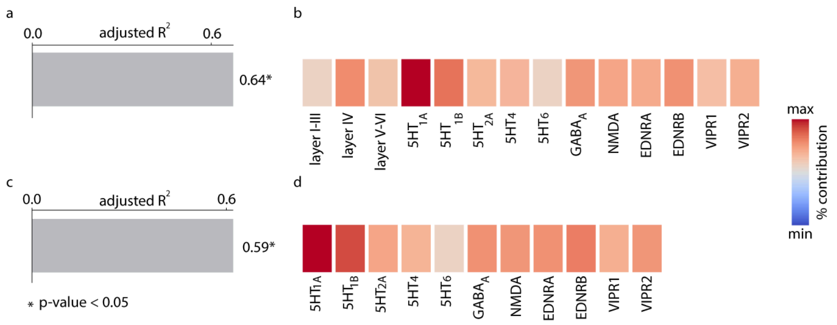

"**FigureS9. Relating perfusion to molecular signaling systems and laminar organization |** We use dominance analysis to assess the contribution of biological features to regional variations in cerebral blood perfusion. Dominance analysis distributes the model's explained variance among the input variables, allowing comparison of their relative contribution to predicting perfusion patterns. (a, b) show results for a model including neurotransmitters, neuropeptides and cortical laminar profiles; and (c, d) show results for a model including only neurotransmitters and neuropeptides. Model fit, expressed as adjusted R^2, reaches to 0.64 for the first model (a) and to 0.59 for the second model (c). Asterisks denote significant model fits (in both cases P_{spin} = 0.0099, N_{spin} = 1,000). The percent contributions of each input variable normalized by the total model fit is shown in (b, d)."

We refer to the added supplementary figure in Figure 3 caption:

"...(c) Comparison of perfusion with maps of neurotransmitter receptors (from PET imaging) and neuropeptide receptors (from transcriptomics) [46, 91]. Colors indicate directions of association (red = positive, blue = negative) and asterisks indicate significant associations. See Fig.S9 for results obtained using a multivariate model with dominance analysis. Methodological details about each neurotransmitter receptor PET map are provided in Table. S3."

9. The authors employed a non-parametric "spin test" to assess spatial correlations. However, this method is specifically designed for cortical surface data and does not accommodate subcortical regions. Given that the current analyses include cortical and subcortical areas, the spin test may not be the most suitable approach. Additionally, the spin test is limited by potential biases introduced when the medial wall—typically lacking data—is rotated into cortical areas (Markello et al., NeuroImage, 2021). An alternative method, such as variogram matching (Burt et al., NeuroImage, 2020), which can handle both cortical and subcortical regions and account for spatial autocorrelation more comprehensively, would be more appropriate for the current study.

We apologize for the confusion - as explained in the response to point 7, we did not include the subcortex in any analysis. As the results are reported for the cortex, we use spin-tests to avoid variogram fitting issues. In a previous benchmarking study, we found that parameterized nulls tend to incur greater false discovery rates compared to non-parametric “spin tests” (Markello & Misic, 2021).

Part of the reason for this result was that parameterized generative models may not perfectly reproduce the spatial autocorrelation structure of empirical maps. To verify this, we used a generative framework (variogram matching; Burt et al., 2020) to generate 1000 surrogate maps, as well as 1000 spin-permuted maps. We then assessed how well each approach preserved the spatial autocorrelation of our empirical perfusion data using Moran's *I*, a metric for quantifying spatial autocorrelation. In the figure below, we show the difference between Moran’s *I* for the empirical data and each of the surrogate populations. Consistent with previous results, we find that the generative model does not reproduce the spatial autocorrelation of the empirical data better than spins. We therefore retain the spin test nulls as the primary method of statistical inference.

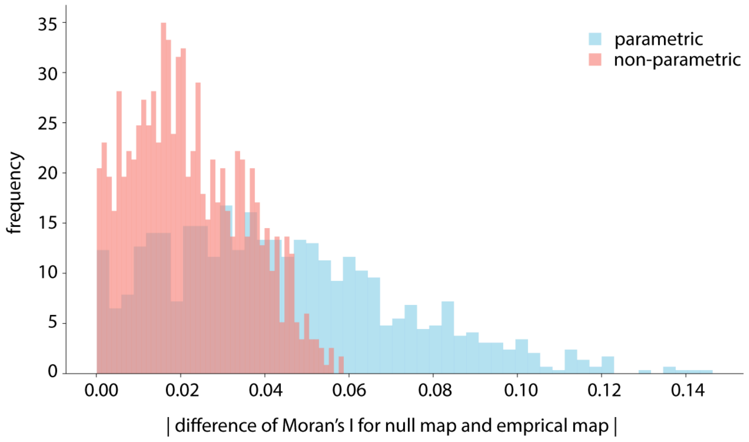

References:

Markello, R. D., & Misic, B. (2021). Comparing spatial null models for brain maps. NeuroImage, 236, 118052.

Burt, J. B., Helmer, M., Shinn, M., Anticevic, A., & Murray, J. D. (2020). Generative modeling of brain maps with spatial autocorrelation. NeuroImage, 220, 117038.
